# Integration of DNA Methylation and Transcriptome Data Improves Complex Trait Prediction in *Hordeum vulgare*

**DOI:** 10.3390/plants11172190

**Published:** 2022-08-24

**Authors:** Pernille Bjarup Hansen, Anja Karine Ruud, Gustavo de los Campos, Marta Malinowska, Istvan Nagy, Simon Fiil Svane, Kristian Thorup-Kristensen, Jens Due Jensen, Lene Krusell, Torben Asp

**Affiliations:** 1 Center for Quantitative Genetics and Genomics, Aarhus University, 4200 Slagelse, Denmark; 2 Departments of Epidemiology & Biostatistics and Statistics & Probability, Institute for Quantitative Health Science and Engineering, Michigan State University, East Lansing, MI 48824, USA; 3 Section for Crop Sciences, Department of Plant and Environmental Sciences, Copenhagen University, 2630 Taastrup, Denmark; 4 Nordic Seed A/S, Grindsnabevej 25, 8300 Odder, Denmark; 5 Sejet Plant Breeding, Nørremarksvej 67, 8700 Horsens, Denmark

**Keywords:** multi-omics prediction of complex traits, barley, DNA methylation, transcriptome, RadiMax semi-field phenomics facility

## Abstract

Whole-genome multi-omics profiles contain valuable information for the characterization and prediction of complex traits in plants. In this study, we evaluate multi-omics models to predict four complex traits in barley (*Hordeum vulgare*); grain yield, thousand kernel weight, protein content, and nitrogen uptake. Genomic, transcriptomic, and DNA methylation data were obtained from 75 spring barley lines tested in the RadiMax semi-field phenomics facility under control and water-scarce treatment. By integrating multi-omics data at genomic, transcriptomic, and DNA methylation regulatory levels, a higher proportion of phenotypic variance was explained (0.72–0.91) than with genomic models alone (0.55–0.86). The correlation between predictions and phenotypes varied from 0.17–0.28 for control plants and 0.23–0.37 for water-scarce plants, and the increase in accuracy was significant for nitrogen uptake and protein content compared to models using genomic information alone. Adding transcriptomic and DNA methylation information to the prediction models explained more of the phenotypic variance attributed to the environment in grain yield and nitrogen uptake. It furthermore explained more of the non-additive genetic effects for thousand kernel weight and protein content. Our results show the feasibility of multi-omics prediction for complex traits in barley.

## 1. Introduction

Barley (*Hordeum vulgare* L.) is the fourth most important cereal crop in world production [1]. The future exploitation of barley depends on its ability to adapt to abiotic and biotic stresses. Grain yield, thousand kernel weight (TKW), grain protein content, and grain nitrogen uptake are some of the agro-economically important, complex traits in barley. Therefore, it is essential to focus on improving these traits to develop new varieties of barley equipped to cope with predicted global environmental changes [2].

Phenotype–genotype relationships are complex and challenging to understand fully—to do so, multiple genome-wide omics approaches can be useful. The relationship between the trait and genetic information involves different regulatory layers, such as epigenetic and transcriptome modifications [3]. The environment in which a crop is grown strongly influences these intermediate regulatory layers. When the plant is exposed to abiotic stress, it reacts quickly at the gene expression level, activating stress response genes [4] and triggering epigenetic changes such as DNA methylation, which can also lead to epigenetic regulation of gene expression [5,6,7]. Epigenetic marks can be tags that alter DNA accessibility and chromatin structure, influencing gene expression and activity [8]. One of these modifications is DNA methylation, which is the addition of a methyl group to one of the four bases, typically cytosine [9]. Cytosine DNA methylation plays a central role in silencing transposons and other repetitive regions as well as regulating gene expression [10]. There is no consensus on the relationship between gene expression and DNA methylation. Research suggests that when DNA methylation occurs within gene promoter regions, it is believed to reduce gene expression [7,11]. On the other hand, when DNA methylation occurs within introns or exons, it is correlated with an increase in gene expression [11]. Nevertheless, it is also suggested that it is the gene expression changes that drive the methylation differences in the regulatory regions more than the other way around [12]. The environment can alter both the transcriptome and epigenome, and their profiles can yield new information on the relationship between line and trait [13]. A complete understanding of DNA methylation and its impact on regulatory regions is still missing. Not even the effect of stress-induced DNA methylation is clear, or the regulatory effect of DNA methylation on gene expression. The readers are recommended to read Richards and Pigliucci [14] for further insight into the complex relationship.

Perhaps progress in understanding complex traits in crops can be achieved by integrating multiple omics such as genomic, transcriptomic, and DNA methylation data in our analysis [15].

It has previously been reported that the use of multi-omics profiles increased the proportion of variance explained and accuracy in the prediction of survival for breast cancer patients compared to using only genomic information [16]. Integrating gene expression information in multi-omics predictions could potentially improve the predictability of complex traits in *Drosophila melanogaster* [17]. For predicting the risk of rheumatoid arthritis, integrating methylation profiles in genomic prediction yielded the best-performing model [18]. The use of multi-omics prediction of plant performance has successfully been applied to predict phenotypic performance in maize breeding [19]. Using metabolomics showed an increase in the predictability of traits in rice [20,21]. In *Arabidopsis thaliana*, it is suggested that DNA methylation data can be useful in the whole-genome prediction of complex traits [22], where epigenetic variation explained 65% of the phenotypic variance using an epi-G kernel. It has been shown that including DNA methylation data in multi-omics prediction models increases the predictability of traits in mammals [23], but this has not yet been demonstrated in plants.

Multi-omics approaches have only been applied to barley in a few studies. Shen et al. [24] investigated ionomic, metabolomic, and proteomic profiles and found several molecular mechanisms related to shoot adaption to salt stress. Ho et al. [25] used transcriptomes, metabolomes, and lipidomes to identify two distinctive salt tolerance mechanisms. Neither of these studies focused on multi-omics prediction. Gemmer et al. [26] did however investigate whether metabolomic prediction could be an alternative to genomic prediction in barley. They concluded that metabolomic prediction alone could not be recommended in barley, but it could be useful in the understanding of complex traits. Our study is the first to investigate the integration of gene expression, DNA methylation, and genomic data for the prediction of traits in barley.

The goal was to (i) investigate the drought effect within gene expression and methylation data, (ii) analyze the mediating effect of gene expression and methylation for treatment on agro-economic traits, and (iii) quantify the relative contributions of SNPs, DNA methylation, and gene expression in multi-omics linear mixed models.

## 2. Results

Seventy-five barley lines were grown in 75 rows under control and water-scarce conditions, one for each line. The rows were grown along a sloped water gradient, with subirrigation from 1.1–3 m below the surface. Furthermore, rain-out shelters were used to induce drought by covering the entire experimental area. During the growth period, sampling for gene expression and DNA methylation was performed. At harvest time, the lines were phenotyped for thousand kernel weight, grain yield, nitrogen uptake, and protein content. Each row was divided into two sub-samples at harvest. One from the deep water scarce-section and one from the shallow control section. The ears were collected, dried, threshed, and weighed for grain measurements, thousand kernel weight and yield. Afterwards, the grain water and protein content was determined, and on the basis of the latter, grain nitrogen uptake too [27].

### 2.1. Descriptive Data Analysis

**Figure 1:**
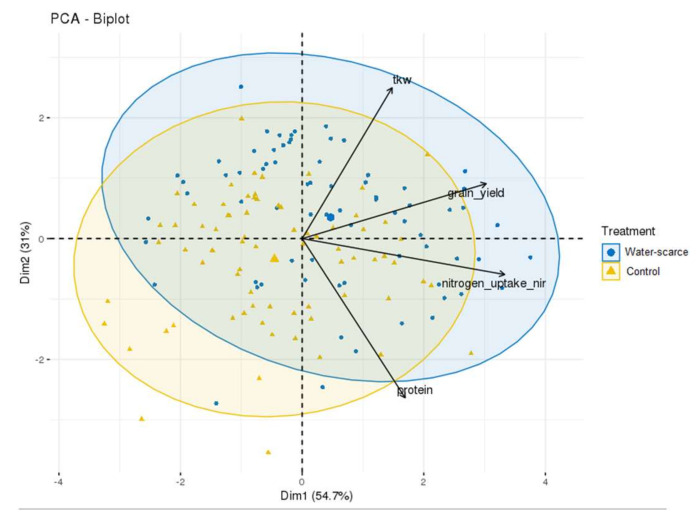
Principal component analysis biplot of the first two principal components based on the 75 lines and their performance within TKW, grain yield, nitrogen uptake, and protein content. Groupings are displayed with a 0.95 concentration ellipse in normal probability, grouped by treatment: water scarce and control. The treatment effect on the lines was examined using principal component analysis (PCA) in R with the packages FactoMineR [28] and factoextra [29], where the scaled traits were used as loadings.

For all traits, the data show an approximately normal distribution (Figure S1A). The variabilities (distribution of random effects) were also normally distributed (Figure S1B). The PCA plots show a trend of lower phenotypic values in the water-scarce samples (Figure 1).

### 2.2. Mediation Analyses

An elaborated association study was performed using the framework of a mediation study [30] on the omics data. The primary objective of the analysis was to evaluate whether the treatment, methylomic, and transcriptomic data had a (direct or indirect) effect on the traits.

**Figure 2:**
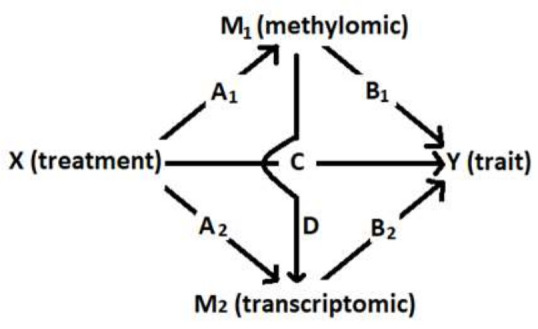
Conceptual framework of the mediation analysis.

Regression models for all of the omics and treatment effects were evaluated using analysis of variance and reporting the *p*-values. An illustrative overview of the mediation paths is given in Figure 2.

Step one in the mediation analysis (see Figure 2, path C) showed β_treatmentC_ was significant for all traits (see Table 1).

**Figure 3:**
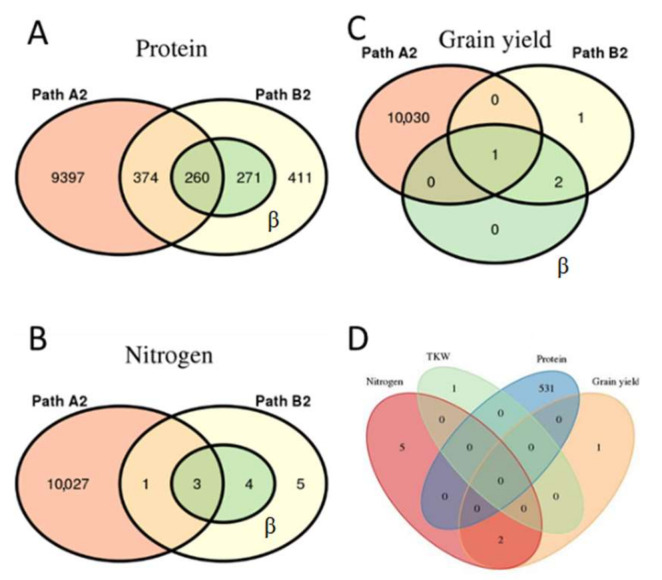
Overlapping significant sites for the different steps in the mediation analysis. Nitrogen is nitrogen uptake. (**A**) Of the 1316 significant sites from path B_2_ on protein, 634 sites overlap with part A_2,_ and 531 of the regression coefficients (β) are lower in absolute value than the beta from path C. Of these 531, 260 are shared with path A_2_ and B_2_. (**B**) Of the 13 significant sites from path B_2_ on nitrogen uptake, 4 of the sites overlap with part A_2,_ and 7 of the β are lower in absolute value than the β from path C. Of these 7, 3 are shared with path A_2_ and B_2_. (**C**) Of the 4 significant sites from path B_2_ on grain yield, 1 of the sites overlaps with part A_2,_ and 3 of the β are lower in absolute value than the β-values from path C. Of these 3, 1 is shared with path A_2_ and 2 with B_2_. (**D**) The relationship between the β values from each trait. Only 2 sites overlap, and it is between grain yield traits and nitrogen uptake. The plots are made using the R package “VennDiagram” [31].

Step two for DNA methylation (path A_1_) showed no significant β-values after correcting for FDR or using the significance level found through the permutation analysis. In step 3 (path B_1_), no significant β-values were found for the four traits, both after FDR correction and after setting a new significance threshold through permutations. The gene expression data set shows that step two (path A_2_) showed 10,031 significant β-values after correcting FDR with BH (Figure S2A). Step three (path B_2_) also showed multiple significant β_mediatorB_ values. For grain yield, there were four significant β-values (Figure S2B), protein had 1316 significant genes (Figure S2C), nitrogen had 13 significant genes (Figure S2D), and TKW had one significant gene (Figure S2E). The number of significant β_treatmentB_ values that were smaller in absolute values than β_treatmentC is_ presented in Table 1, which represents the number of mediating sites for each trait. The results from the mediation analysis were compared over the different paths within traits and are presented in Figure 3A–C.

The relationships between the regression coefficients from each trait were also compared. Only two sites were found to overlap, and these were between the traits of grain yield and nitrogen uptake (Figure 3D).

### 2.3. Differential Expression Analysis

**Figure 4:**
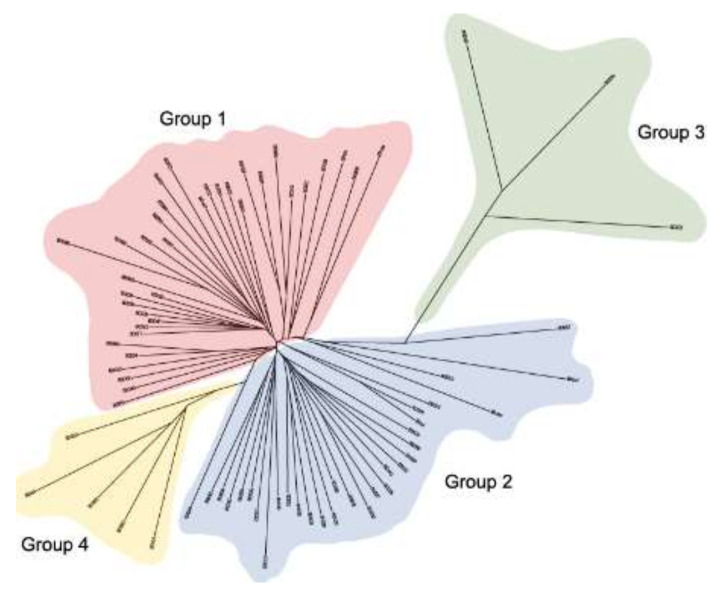
Dendrogram of the 75 lines based on all SNPs in the transcriptome. The 75 lines are divided into four groups indicated with the colors red (group 1), blue (group 2), green (group 3), and yellow (group 4).

Lines were assigned to four groups based on the dendrogram (Figure 4) calculated from the 1,316,898 SNPs detected in the transcriptome.

The DE analysis identified 74 genes that were DE in all the groups when comparing control with the water-scarce treatment (Figure S3).

Additionally, many genes were differentially expressed in at least two groups, ranging from 97 to 218 (Figure S3). Of the 74 genes, six were also significant in path A_2_ in the mediation analysis.

Analysis of Pfam domains in the 74 DE genes identified several domains connected to abiotic stress responses. These included myb-like DNA binding domains, dehydrin, and AP2 (Table S3). In addition, three significantly enriched GO-terms, classified as biological functions, were identified by GO-term enrichment analysis using GOATOOLS.

### 2.4. Multi-Omics Modeling and Cross-Validation

We defined a sequence of seven Bayesian models to perform multi-omics prediction on the four phenotypes. Each phenotype was regressed on treatment plus various combinations of the omics; genomics, transcriptomic, and DNA methylation. After fitting the models, we estimated the proportion of the phenotypic variance of each trait explained by treatment and by each of the omics. To quantify the ability of each of the seven models to predict phenotypes, we implemented a five-fold cross-validation with lines randomly assigned to fold.

**Figure 5:**
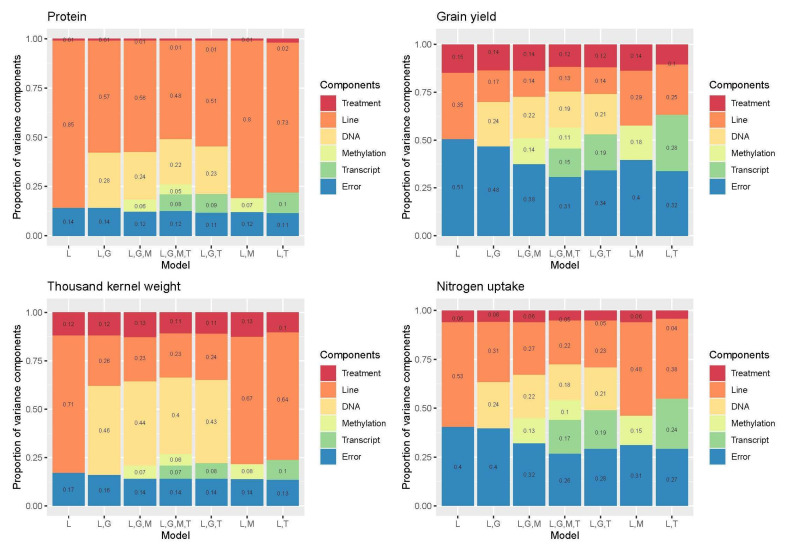
The proportion of phenotypic variance explained by treatment, line, and each of the omics by model. For each of the four traits, the proportion of variance explained by each component in the model is illustrated. Models: L = treatment + line, G = genomic SNP, M = DNA methylation, T= transcriptomic.

For all the traits, the multi-omics model (M_L,G,M,T_) with genomic, DNA methylation, and gene expression information included explained the most variance 0.72–0.92 (Table S4). The proportion of variance explained (PV^2^) by each of the factors included in the model by trait is presented in Figure 5.

**Figure 6:**
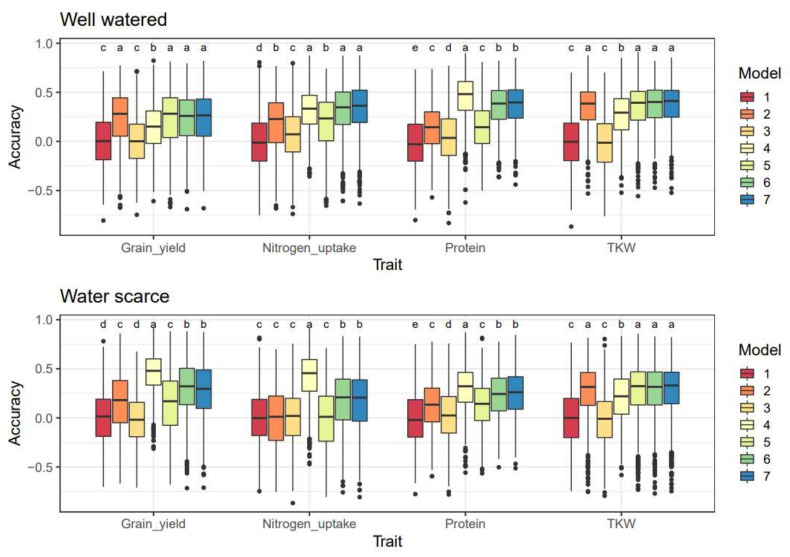
Accuracies for the models 1–7 for each trait by treatment. Ad hoc Tukey tests on Fisher’s transformed correlation group results are indicated as letters above each model. 1 = M_L_, 2 = M_L,G_, 3 = M_L,M_, 4 = M_L,T_, 5 = M_L,G,M_, 6 = M_L,G,T_, 7 = M_L,G,M,T_.

Summing up the performance of M_L,G,M,T_, all PV^2^ apart from DNA methylation, were generally high (Table S4). Genomic information explained 18–40% of the PV^2^. 5–11% of the PV^2^ was explained by DNA methylation and 7–17% by gene expression. In total, all three omics explained 35–53% of the PV^2^. The PV^2^ by gene expression and DNA methylation grouped the traits into two categories, with grain yield and nitrogen uptake with high values and protein content and TKW with low values. The prediction accuracy between the two treatments differed (Figure 6). The accuracy of M_L,G_ and M_L,G,M_ for predicting grain nitrogen uptake was zero in both cases for water scarcity, and 0.18 and 0.19, respectively, for well-watered. In general, for nitrogen uptake, only M_L,T_ gave high prediction accuracy. For grain yield, TKW, and protein, the accuracy under water scarcity was generally lower for the majority of the models.

There is a clear pattern of covariance between the omics. For example, for grain yield in M_L,G,M_, DNA methylation explained 0.14%, which is less than in M_L,M_ due to covariance between DNA methylation and SNPs. The same can be seen in M_L,G,T_, between gene expression and genomic SNPs. Including all three omics as in M_L,G,M,T_, a reduction in the variance explained in all omics is observed. A similar trend can also be observed for the other traits; however, it is less prominent for protein and TKW than grain yield and nitrogen uptake.

## 3. Discussion

In this study, we investigated and analyzed methylation and gene expression in barley under water-scarce and control conditions to integrate these data into multi-omics models. Our results showed that at the time of sampling, we could detect a mild effect on gene expression and none on DNA methylation. Nonetheless, integrating these omics in prediction models improved PV^2^ in all traits. Including more omics in the prediction models improved predictability, but due to covariance, the multi-omics models do not gain the full potential of the individual omics. To strengthen the conclusions, a larger replicated data set with stronger treatment effect would be needed.

### 3.1. Mediating Effect of Gene Expression

In the mediation analysis, the treatment effect could be detected in some genes of the gene expression data (Table 1). The mediation analysis was based on an association study of features covering the entire genome and not only QTLs and eQTLs [32]. Therefore, it was not expected that the entire data set would work as a mediator. As there was no significant mediation effect of DNA methylation, a two mediator analysis was not performed. No sites were significant for DNA methylation, and for gene expression, only a small fraction of the data set was significant. The significant genes in the mediation analyses showed that few genes overlapped in the different steps within each trait (Figure 3A–C). If the treatment effect had been more pronounced, possibly more mediating sites would have been detectable in both omics, as severe drought strongly affects barley performance [33].

In conclusion, as expected, some genes in the gene expression data can be described as mediators. Even though we do not observe complete mediation in gene expression or DNA methylation, we cannot reject the hypothesis that full mediation cannot be found in gene expression or DNA methylation due to a lack of power and limitations in the data set.

### 3.2. Differentially Expressed Genes between Treatments

As the mediation analysis of the gene expression data showed an effect for some genes regarding the treatment effect, this data set was investigated further. A differential gene expression analysis identified 74 differentially expressed genes between the treatments common for the four groups (Figure S3). These results support the result of the mediation analysis, which indicated a treatment effect. Six genes overlapped between the DE and the 10,031 significant genes identified in the mediation analysis. Among the annotated motifs in the DE genes, three were of particular interest: Myb-like DNA binding domains, which are transcription factors known to affect drought tolerance in potatoes [34,35]. They are further discussed in Roy [36] as triggering coordinated changes in gene expression as a response to abiotic stress and with a putative role in epigenetic regulation. Dehydrin and AP2-domains, are known to be key regulators of networks in abiotic stress responses [37] (see Table S3). Dehydrin is a multi-family of proteins produced in plants to respond to both cold and drought stress [38,39]. AP2 domain plays a role in hormone regulation [40] and is involved in seed and flower development. Overexpression of DBF1, which is a member of the AP2 family, resulted in higher tolerance to osmotic stress. The GO-term enrichment analysis identified three significantly enriched motives related to oxidoreductase and dioxygenase activity and known promoters for ABA biosynthesis under drought stress [41,42].

### 3.3. Multi-Omics Prediction Models Improve Predictability

Both gene expression and DNA methylation helped improve the predictability of traits (Table S4). Including more omics in the prediction models improved predictability, but due to covariance, the multi-omics models do not gain the full potential of the individual omics. Based on the analyses of variance and the accuracy of the prediction models (Table S4), gene expression data appears to explain more of the variance than DNA methylation. Adding both DNA methylation and gene expression data (M_L,G,M,T_) increased the variance explained more than when only gene expression data (M_L,T_ and M_L,G,T_) was included. Westhues et al. [19] found that, as in our study, gene expression data increased predictability and was even the best predictor for some traits in T0 maize hybrids. In their models, combined genomic and gene expression data showed robust predictive abilities, but combining these with metabolomics data did not improve predictability. In our case, DNA methylation data (M_L,M_, M_L,G,M_, and M_L,G,T_) did improve predictability, as suggested by Hu et al. [22] and Forno and Celedón [13]. Our DNA methylation data was also not discretized as in [22], which allowed us to retain more information. This was possible because the epi-G kernel (used by Hu et al. [22]) mimics a genomic relationship matrix and therefore loses information, which was unnecessary in our case as we already had a relationship matrix based on genomic information.

Omics delivered a high PV^2^ for all traits. Traits in barley with high PV^2^ are predicted with higher accuracy using Bayesian methods [43]. For this reason, BGLR was an appropriate choice for our data. We relied on the results from the CV, the accuracy of the prediction models, and ad hoc tests of these (Table S4 and Figure 6) to determine the best prediction model [44]. Furthermore, we found that combining the data from the two treatments decreased prediction accuracy (data not presented), as also shown in Lorenz et al. [45]. In agreement with Lorenz et al. [45] and Nielsen et al. [46], the treatments used could not be used to predict the performance of plants in the other treatment group. Therefore, we chose to evaluate accuracies within the treatments and considered this the best approach.

When examining the distribution of the explained variance (Table S4 and Figure 5), the model that explained the most variance for all traits included all omics; genomic, DNA methylation, and gene expression information. This is in contrast to Westhues et al. [19] but in agreement with the findings of Vazquez et al. [16], Guo et al. [47], and Wang et al. [21]. In M_L,M_, M_L,G,M_, and M_L,G,M,T_ a small effect on the explanatory power from including DNA methylation can be seen, and a stronger effect from adding gene expression can be seen in M_L,T_, M_L,G,T_, and M_L,G,M,T_. The DNA methylation data set explained between 0.08–0.18 of the variance in the models, which is lower than the 0.65 found in previous studies of Arabidopsis [22]. The Arabidopsis epiRIL population structure in Hu et al. [22] differed from the barley structure in our study, as the population was derived from two parents, one with a mutation in *DDM1*, therefore only expected to differ in methylation. For this reason, their offspring are segregated in a more definite pattern than the barley in our population, which is highly homozygous inbred or DH lines and commercial cultivars with varying degrees of relatedness.

There is a presence of covariances between the omics. However, it is only in the grain yield that a positive covariance was found when combining genomics with either DNA methylation (M_L,G,M_) or gene expression data (M_L,G,T_) (Table S4). Here the combination of genomic and gene expression data (M_L,G,T_) increased the variance explained. In all other traits, the variance explained is the same as with only one omic data set (M_L,G_, M_L,M_, M_L,T_) or even less as in nitrogen uptake and TKW, where gene expression data explains more variance without the addition of genomic data (M_L,T_). Nevertheless, adding the three layers of omics together (M_L,G,M,T_ in Table S4) explained the highest PV^2^ of all the models for grain yield, TKW, and nitrogen uptake. Only for protein content was M_L,T_ the most accurate. In this model, most of the variance is explained by the line effect, which is undesired with genomic prediction. In genomic prediction, you want the genomic information to explain more in order to rely on your SNPs. Therefore, M_L,G,M,T_ was still the best. Selecting M_L,G,M,T_ as the best was based on the high PV^2^ of the traits in the model, as high PV^2^ gives higher prediction accuracy [48,49]. In Wang et al. [21], the inclusion of gene expression data did not improve predictability in hybrid rice for yield, TKW, number of grains per panicle, or number of tillers per plant, but instead, predictability decreased. Our findings show the opposite for protein and nitrogen uptake in barley, agreeing with studies in maize [19].

### 3.4. The Genetic Complexity of the Traits and Treatment Effect on Multi-Omics Prediction

The traits investigated have been evaluated based on genomic, transcriptomic, and DNA methylation data. These data show a difference in the genetic complexity between protein and TKW compared to grain yield and nitrogen uptake. The mediation analyses showed that only nitrogen uptake and grain yield have overlapping mediating genes (Figure 3D). These two traits also showed similar results in the predictions because the measurements for nitrogen uptake are profoundly affected by yield and estimated based on the grain protein content [27]. Furthermore, there were seasonal differences in the nitrogen level in the RadiMax facility in 2017 [27]. PV^2^ delivered by genomic information is higher for protein and TKW traits, and little PV^2^ is explained by the environment or residual error for these traits. Therefore, these traits were less affected by the environment, as the environmental effect indirectly decreases the PV^2^ explained [50]. In rye (*Secale cereal* L.), triticale (×*Triticosecale* Wittmack), and durum wheat (*Triticum durum* Desf.), TKW was found to be controlled by multiple major QTL in contrast to grain yield [51,52,53]. Nitrogen uptake in grains is highly affected by the growing conditions of the experimental setup, as shown by Pan et al. [54] in wheat. This supports the conclusion that nitrogen uptake is a complex trait in cereals. A study in wheat has revealed seven additive QTL accounting for 43.45% of the protein content phenotypic variance [55]. In wild emmer wheat (*Tritium turgidum* ssp. *dicoccoides*), multiple QTL for both TKW and protein content were found [56]. Adding more omics layers explained more of the variance in grain yield and nitrogen uptake in this species. Modeling performance of wild emmer wheat showed that PV^2^ of these traits were low, and the residual errors were high. The large nitrogen effect can again explain the high residuals in our study from the previous season and the large effect seen in protein content. This suggests that for TKW and protein, the variance of the line is highly affected by the nitrogen effect [57], and our omics data sets do not capture this environmental effect. It would be interesting to see whether this phenomenon could be captured using metabolomics as in rice [20,21]. Benešová et al. [58] demonstrated that the effect of drought in maize could be captured using proteomics. Alternatively, it would be of interest to determine if the trend would be the same in another experimental setup, such as one with a severe drought effect or other types of abiotic stress.

Besides PV^2^ by genomic information, we also calculated the PV^2^ by DNA methylation and gene expression. Previous studies [17,59,60,61] have used the term heritability to refer to the proportion of variance of a phenotype explained by gene expression. However, gene expression is a trait and is not fully heritable. Therefore, we refer to the parameter simply as the proportion of variance explained by gene expression profiles, which summarize all the genetic and non-genetic factors that affect gene expression and how this translates to variation in other phenotypes.

PV^2^ by DNA methylation was found to be low for all traits. In contrast, the PV^2^ by gene expression was high and showed similar values for all traits of between 7–17%. It was previously suggested that the PV^2^ by gene expression could be used to understand the hidden heritability due to poorly tagged variants in the genotyped SNPs [59]. Our results (Table S4) clearly show that gene expression explains variance that the DNA information does not capture in the M_L,T_ models. The PV^2^ by gene expression are all considered high and can partly be explained by (i) the variation within the expression profiles of the lines, which explains some of the variation from the lines (see Table S4; M_L_ compared to M_L,T_); (ii) gene expression was transformed into an expression similarity matrix based on the principal components, thus reducing noise from individual expression profiles [60]; and (iii) impact on complex traits. The PV^2^ and the overall results from the gene expression data show that there are biologically meaningful genes that should be investigated further. This could be achieved with TWAS [62]. Kremlin et al. [61] state that the more complex a trait, the more TWAS outperformed GWAS, and the combination of both increased the power of gene detection. eQTL analyses [63] or even GxEMM [64], could be other possibilities if more environments are included. As Li et al. [17] suggested, including gene expression data in our prediction models improved predictability; in fact, including both DNA methylation and gene expression data improved predictability and resulted in high PV^2^ for all traits.

The DNA methylation data increased the proportion of variance explained in grain yield and nitrogen uptake (M_L,M_; M_L,G,M_; and M_L,G,M,T_; Table S4 and Figure 5). We expected a change in DNA methylation between the two treatments [65]. Epigenetic regulations may lead to the downregulation of gene expression [66]. However, in our data, 67 of the 74 DE genes were upregulated (data not shown). The treatment, therefore, had a more profound effect on gene expression than on DNA methylation in our samples. DNA methylation can be influenced by substitution mutations, which appear as epimutations [67].

Another potential explanation for the lack of power in the DNA methylation data is that it could have been due to the development stage of the plants at the time they were sampled. If DNA methylation affects gene expression, it is rarely seen as a general decrease in gene expression [13]. Its activity is local, and it would be beneficial to look at the methylation profile more closely, e.g., with the whole-genome methylation analysis. The most striking differences in DNA methylation levels are usually between different tissues and developmental stages [65,68]. At the time of sampling, spring barley in early July in Denmark will have passed the flowering stage and be at an early maturing stage (~BBCH 70) of seed development. Hence a later sampling time would have been preferred.

Lastly, the lack of power in our models could be explained by the small population size and late drought application. Only 75 samples were collected for each treatment compared to 505 in Hu et al. [22] and 502 in Bernal Rubio et al. [23]. The application of drought only took place one month before sampling, and due to sufficient available water in the soil, the onset of the drought symptoms was delayed [27]. Higher evapotranspiration had been expected, but the season in 2017 was cold and cloudy [27]. Therefore, due to noise and a lack of power, we cannot reject the hypothesis that the water-scarce treatment in the experiment affected the DNA methylation level.

### 3.5. Perspectives for Multi-Omics Prediction in Breeding Programs

It is possible to predict traits accurately with a small training population (TP) (*n* = 200) without sacrificing accuracy [43,45,46,69]. Our population was small, 75 within each treatment, which meant that we lost accuracy, as predicted by Nielsen et al. [46]. In the future, increasing TP is expected to increase the accuracy of the models.

Depending on trait and cost, it is crucial to look at the distribution of the variance explained and the accuracy of a model. When it comes to TKW, the distribution of variance explained and the accuracy appears to be good enough only with genomic SNP data (M_L,G_), whereas, for grain yield and nitrogen, all three omics (M_L,G,M,T_) would be the best options. For protein, genomic and gene expression information (M_L,G,T_) would be sufficient for a good prediction. The gene expression and DNA methylation data only describe the environment and time-point from which they were sampled. The choice of omics for each trait appears to be the same for both treatments, as the Tukey test in Figure 6 shows no significant difference in the accuracies between the best models within each treatment. The TP should be maintained and optimized with information from recent breeding cycles to keep a high prediction accuracy [70].

The traits are also selected with different criteria depending on the use. Large kernels with low nitrogen content are, e.g., desired for malting [71], while there is less selection in fodder types. Generally, all lines are selected for high yield [72].

This paper shows how it can be beneficial to include different omics data sets in models predicting different phenotypes for different complex traits in barley. Depending on the trait of interest, different combinations of omics data should be used in the prediction model to increase predictability. The results indicate that in barley, (1) mild drought can be detected in gene expression sampled from the flag leaf, (2) including DNA methylation and gene expression data increases predictability for grain yield and nitrogen uptake, (3) using only genomic SNPs is sufficient for TKW, and (4) both genomic and transcriptome SNPs for protein gives the best prediction model.

## 4. Materials and Methods

### 4.1. Plant Material and Data Collection

Plants were grown in RadiMax, a state-of-the-art phenomics screening facility at the University of Copenhagen, Højbakkegaard, Taastrup, Denmark. The RadiMax facility consists of four units forming individual planting beds with the dimensions of 9.7 × 40 m. Plants were grown in rows along a sloped water stress gradient created by a subsurface-irrigation system with 10 levels and movable rainout shelters. The barley experiment was made in unit two of the facility, with a soil depth increasing from 1.1 m at the borders to a maximum of 3.0 m at the center. Induction of drought was performed by covering the unit with a rainout shelter. A more detailed description of the facility can be found in Svane et al. [27], and a cross-section and field layout are provided in Figure S4.

Seventy-five lines of spring barley were grown in individual rows on the south side of unit two in the RadiMax facility in 2017. The experiment was one block of the randomized complete block design described in Svane et al. [27], where the lines were seeded with a 25 cm row distance in one replicate. The drought effect was not possible to randomize and the block was split in two by dividing all rows into two treatments, water-scarce (north) and control (south); see Figure S4. The majority of the plant material came from advanced breeding lines provided by the Danish breeding companies Nordic Seed and Sejet Plant Breeding, combined with one cultivar and three landraces; see Svane et al. [73]. Water-scarce conditions were induced by rainout shelters covering the entire experimental unit from 7 June 2017, leaving plants to take up water from the soil and the sloped subsurface irrigation system.

Leaf tissue was collected in bulk from five flag leaves at the grain filling stage (5 July 2017). Two samples were collected from each line, 40 cm from the border of the RadiMax facility (control) and 40 cm from the centerline (water-scarce). This defined the two treatments used in the multi-omics prediction models. Phenotypic data collection was performed as described by Svane et al. (2019). Due to practical reasons and limitations in the harvesting tool, each row was divided into two sub-samples. One from the deep water-scarce section and one from the shallow control. The barley ears were collected in bulk, dried, threshed, and weighed to measure grain yield (mg ha^−1^) and TKW (g). Grain protein content (% of dry matter) was determined by near-infrared transmission measurements (Infratec grain analyzer, Foss, Hillerød, Denmark). Grain nitrogen uptake (g N m^−2^) was estimated based on the grain protein content, as described in ISO 16634-2:2016 [74]. For more specific details, see Svane et al. [27].

For a given trait y, a linear mixed model was used to investigate normality and variability:

$$y_{ij}=T_{j}t+l_{i}+e_{ij}$$

where $y_{ij}$ is the phenotypic observation of line i and treatment $j=\left\{1,2\right\}$, $T_{j}$ is dummy variable indicating if the treatment is water-scarce or control, t is the treatment effects, $l_{i}\sim N\left(0,\sigma_{l}^{2}\right)$ is the random effect of line i and the variance $\sigma_{l}^{2}$, $e_{ij}\sim N\left(0,\sigma_{e}^{2}\right)$ is the independent and Gaussian distributed residuals with the variance $\sigma_{e}^{2}$. The above model was estimated by REML using the “lme4” package [75] in R [76].

### 4.2. Genomic and DNA Methylation Data

The epiGBS approach [77] was used for reduced representation bisulfite sequencing. Isolated genomic DNA (400 ng) was double digested using PacI and NsiI and subsequently ligated with methylated adaptors. Equal volumes of 12 ligates were pooled and subsequently purified and size-selected using AMPureXP beads (Beckman Coulter, Inc., Indianapolis, IN, USA) and finally subjected to nick translation with DNA polymerase I (NEB) and 5-methylcytosine dNTP mix (Zymo Research, Freiburg im Breisgau, Germany). Bisulfite conversion on each pool of 12 samples was performed with an EZ DNA Methylation Lightning™ Kit (Zymo Research, Freiburg im Breisgau, Germany). Libraries were amplified with the KAPA Uracil Hotstart Ready Mix, followed by another round of purification and size selection. Paired-end 150 bp sequencing was performed on four lanes of an Illumina HiSeq 4000 platform by Novogene Ltd. (Beijing, China).

The paired-end reads were processed following the epiGBS pipeline [77]; (https://github.com/thomasvangurp/epiGBS (accessed on 6 February 2018)) to demultiplex the samples, trim adapters, remove PCR duplicates, and assemble the de novo reference sequences. The epiGBS methylation_calling.py script was used to distinguish and split genetic and epigenetic variation simultaneously. The resulting variants were separated into two files: one for genetic polymorphisms and the other for methylation polymorphisms. SNPs in high linkage disequilibrium (R^2^ > 0.9) and more than 20% of missing values were filtered out, leaving a set of 7014 polymorphic SNPs. Raw methylation data were filtered based on a minimum of 5X read coverage, and only positions present in at least 80% of the samples were considered for analysis. Methylation levels were calculated as the number of reads with a methylated position divided by the total number of reads matching that same position. Methylation sites with high proportions of zero and “NA” states were filtered out. Afterwards, methylation sites were divided into three DNA base sequence contexts: CG (cytosine and guanine), CHH, or CHG (H corresponds to adenine, cytosine, or thymine).

### 4.3. Barley Reference Genome Modifications

A split version of the barley reference genome IBSCv2 was used [78]. Briefly, a custom script split the pseudo chromosomes at the center to comply with the 512 Mb size limit of bai indexing by samtools [79], and the split chromosome model was used as a reference throughout the experiments. Another custom script was used to recalculate back to the original coordinates.

### 4.4. RNA Extraction and RNA Sequencing

Total RNA was extracted using the Total Plant RNA kit (Sigma-Aldrich, Schnelldorf, Germany). RNASeq library construction and sequencing were performed by the Beijing Genomics Institute (BGI). Paired-end sequencing was performed on the Illumina HiSeq 4000 platform (2 × 100 bp, about 20 M reads per sample).

### 4.5. Quality Control, Short-Read Alignment, and Expression Quantification

Reads were quality trimmed and adapters removed by Trimmomatic [80], and the trimmed RNA-Seq reads were mapped to the barley reference genome using HISAT2 [81]. Gene-based counts and transcript-based normalized read counts (per kilobase million, TPM) were quantified by StringTie [82]. Raw data was extracted from the transcript abundance files by the prepDE.py script of the StringTie package.

### 4.6. Mediation Analysis

A mediation analysis [30] was performed using a linear mixed model approach to investigate the genetic information one feature at a time. DNA methylation and gene expression data were treated as two separate mediators [83]. DNA methylation was investigated both within each type of methylation and for all types combined. The normalized reads in the gene expression data, i.e., the TPM values of all the samples, were filtered so that transcripts with median value zero and average TPM < 1 were excluded. An overview of the theoretical mediation paths is given in Figure 2.

An overview of the response and predictor variables (fixed and random) within the different regression models are given in Table S1. When investigating mediation, there are three potential causal relationships (illustrated in Figure 2): 1—path C, the independent variable (treatment) affects the dependent variable (here trait). 2—paths A_1_ and A_2_, the independent variable (treatment) also affects the mediator variable (DNA methylation or gene expression). Path 3—B_1_ and B_2_, the mediator (DNA methylation or gene expression), then affects the dependent variable (trait). It is also possible that there are two mediators, where one affects the other, path D.

If the regression coefficient (β-value) for treatment in path C is not significant, the treatment cannot predict the trait and, therefore, cannot be a predictor of DNA methylation or gene expression either. When no association between the mediator (DNA methylation or gene expression) and the independent variable (treatment) is found in path B, it cannot be a mediator either. The β-value for treatment in path B should be significant and smaller in absolute value than C.

The regression coefficients of the mediator models were evaluated through an analysis of variance (ANOVA); here, the *p*-values are reported. The *p*-values were corrected for a false discovery rate (FDR) of 0.05 using two approaches; Benjamini and Hochberg (BH) [84] and permutation analysis (PA). For PA, the permutations ran 1000 times, resampling each time, and the smallest *p*-value from each permutation was collected. The relationships between the different paths and traits were investigated for overlapping sites.

### 4.7. Differential Gene Expression Analysis

The gene counts from StringTie were used for differential gene expression (DE) analysis with DESeq2 [85]. Raw count data were normalized using the default settings in DESeq2 [85]. Differentially expressed (DE) genes were identified by using generalized linear models in DESeq2, where initially, all lines from one treatment were treated as “pseudo-replicates” and compared against all lines from the other treatment.

Due to the relatively low effect of treatment compared to line effects, groups were defined based on genetic relatedness for the DE analysis. SNP variants were called from the bam alignments from the mapped RNA-Seq reads using Freebayes [86].

A pair-wise distance matrix for the 75 lines within each treatment was calculated from the (unfiltered) vcf file using the software VCF2dis (https://github.com/BGI-shenzhen/VCF2Dis (accessed on 1 April 2018). The distance matrix was used as input for the Neighbor program of the PHYLIP package (version 3.697, Felsenstein, 2003) to generate a dendrogram using the Neighbor-joining method.

Genes were considered significantly differentially expressed, based on a two-fold change in expression between treatments and an FDR ≤ 0.01.

### 4.8. GO-Term Enrichment Analysis

Gene ontology (GO) term enrichment analysis was conducted using GOATOOLS [87]. GO-terms that were statistically over-represented among the differentially expressed genes compared to the complete gene set were corrected for a false discovery rate FDR of 0.05 using the BH method [84].

### 4.9. Integrating Multi-Omics Data Using Bayesian Models

Bayesian models previously described in Vazquez et al. 2016 [16] were used for multi-omics prediction. Briefly, each phenotype was regressed on treatment (treated as a “fixed” effect) plus omics using a linear model of the form:

(1) $$y_{ij}=\mu_{j}+L_{i}+u_{i}+g_{ij}+m_{ij}+\varepsilon_{ij}$$

where $y_{ij}$ is a phenotype (separate models were fitted to grain yield, TKW, protein, and nitrogen uptake) of line *i* under treatment *j*, $\mu_{j}$ is the mean of irrigation treatment *j* (*j* = 1,2), $L_{i}\begin{array}{c} iid\\ \sim \end{array}N\left(0,\sigma_{L}^{2}\right)$ is the random effect of the line, $u=\{u_{i}\}\sim MVN\left(0,K_{SNP}\sigma_{u}^{2}\right)$, $g=\{g_{ij}\}\sim MVN\left(0,K_{GE}\sigma_{g}^{2}\right)$, and $m=\{m_{ij}\}\sim MVN\left(0,K_{M}\sigma_{n}^{2}\right)$ are random effects representing the regression of the phenotype on SNPs, gene expression, and DNA methylation data, $K_{SNP}$, $K_{GE}$, and $K_{M}$ are similarity matrices derived from centered-scaled SNPs, gene expression, and DNA methylation data. For instance, $K_{SNP}$ is an additive-genomic relationship matrix derived from the SNPs, and $\sigma_{u}^{2}$, $\sigma_{g}^{2}$, and $\sigma_{n}^{2}$ are variance parameters associated with SNPs, gene expression, and methylation, respectively. The normalized reads from the gene expression were filtered to exclude transcripts with median value zero and average TPM < 1. The genomic SNPs were obtained from the methylation GBS data.

The genomic, gene expression, and DNA methylation relationship matrices (***K***.) were derived using the getG() function of the BGData R-package [88]. The linear mixed models were fitted using the BGLR R-package [89]. The treatment means were assigned flat priors (i.e., fixed effects), and variance parameters were assigned scaled-inverse chi-square priors with the default parameters for the prior scale and prior degrees of freedom (5, see Pérez and de los Campos [89] for further details).

We defined a sequence of seven models by including combinations of the omic-effects in model [1] presented earlier. M_L_ was our baseline model and included only treatment (TRT) and line (L) effects. We then expanded this model by adding omic information: M_L,G_ = TRT + L + SNPs (genomic SNPs), M_L,M_ = TRT + L + ME (DNA methylation), M_L,T_ = TRT + L + T (transcriptomics), M_L,G,M_ = TRT + L + SNPs + ME, M_L,G,T_ = TRT + L + SNPs + T, M_L,G,M,T_ = TRT + L + SNPs + ME + T. An overview is given in Table S2 and scripts can be found in Supplementary File S1.

We first fitted the models to the entire data set and used the samples from the posterior distribution of those models to estimate the proportion of the phenotypic variance of each trait explained by treatment and by each of the omics. The proportion of variance explained (PV^2^) by each term in the model was estimated using the methods described by Lehermeier et al. [90], which fully account for the covariances between features caused by LD (e.g., SNPs, transcripts, methylation sites) within each omics set. This is carried out by using principal component analysis to remove population structure from the phenotypic variance. The proportion of each variance component that contributed to the total phenotypic variance among the lines was used to estimate phenotypic variance expressed as relative variance components. The proportion of total phenotypic variance explained by the genomic information corresponds to narrow-sense heritability ($\hat{h^{2}}$) [57,90]. Genomic variance is equivalent to the genetic variance defined in quantitative genetics for multiple QTL [91].

To quantify the ability of each of the seven models to predict phenotypes, we implemented a five-fold CV with lines randomly assigned to folds. Therefore, when predicting the phenotypes of a line in either well-watered or water-scarce conditions, no omics data for a line in the testing set was used when training the model. The assignment of lines to folds was entirely at random, and we replicated the 5-fold CV 200 times. From each CV, we computed Pearson correlations between predicted and observed phenotypes. Our estimate of prediction accuracy for each model was the average correlation by model-trait combination across the 200 CVs. To compare the prediction accuracy of each of the models, we conducted post hoc analyses using Tukey Honest-Significant-Difference tests applied to Fisher’s transform of the correlations; these analyses were implemented using the R-package “Agricolae” [92]. The script is available in Supplementary File S2.

## Figures and Tables

**Table 1:** β-value from step one in the mediation analysis, path C, and the number of significant β-values from step 3 path b_2_ are lower in absolute value than the absolute value of the β-value from path C.

	Path C	No. of Mediating Sites
Trait	β-value_C_	|β-value_B2_ | < |(β-value_C_|
Grain yield	−0.78 ***	3 *
TKW	−2.99 ***	1 *
Nitrogen uptake	−9.87 ***	7 *
Protein	0.17 ***	531 *

* *p*-value <0.05, *** *p*-value < 0.001. The β-values in path B_2_ are mean values for all the significant β-values.

## Data Availability

Raw data for the DNA methylation can be found in methylation.bed, for transcriptomic data in transcripts.csv, genomic information in genotypes.MAF2 and phenotypic information in phenotypes.txt.

## Supplementary Materials

The following supporting information can be downloaded at: https://www.mdpi.com/article/10.3390/plants11172190/s1, Scripts for the multi-omics integration are available in Supplementary File S1 and the cross-validation in Supplementary File S2.
