# Supplementary material for: Integration of DNA Methylation and Transcriptome Data Improves Complex Trait Prediction in Hordeum vulgare
Source: Plants (Basel). 2022 Aug 24;11(17):2190. doi: 10.3390/plants11172190 (PMC9459846; doi:10.3390/plants11172190)
Supplement: Supplementary file 1 [file plants-11-02190-s001.zip › File S1.PDF]

ANOVA

Pernille-Gustavo

8/9/2018

Variance Components Estimates

Parameters

```
## Parameters
 maxPropNA=0.05
 maxPropZeros=0.9
 maxPropOnes=1
 trait="nitrogen_uptake_nir" # 'grain_yield'  'protein'   'tkw' 'nitrogen_uptake_nir'
 nIter=12000
 burnIn=2000
 thin=2
 verbose=F #T/F
##
```

Loading the data

```
path='/mnt/research/quantgen/projects/pernille'
setwd(paste(path,'/TEST/output',sep=''))
library(BGLR);library(BGData)
load('../../PREPARATION/output/PREP.RData')
y=scale(TRAIT[,trait])
```

Folders

```
dir.create(paste0('ANOVA'))
setwd(paste0('ANOVA'))
dir.create(trait)
setwd(trait)
```

ANOVA-Table

```
VAR=matrix(nrow=10,ncol=7,NA)
rownames(VAR)=c('M1','M2','M3', 'M4','M5','M6', 'M7','M8','M9', 'M10')
colnames(VAR)=c('TRT', 'Line','SNP','MET', 'DE', 'Model','Error')
VAR.LOW=VAR
VAR.UP=VAR
```

Models

The models we fitted include the effects of the experimental design (treatment, row and column) plus the effects of SNPs, Methylation, and Gene Expression, plus interactions. We combined these factors to obtain the following sequence of models

	Line	SNP	Methylation	Transcript expression
M1	X	-	-	-
M2	X	X	-	-
M3	-	X	-	-
M4	X	-	X	-
M5	-	-	X	-
M6	X	-	-	X
M7	-	-	-	X
M8	X	X	X	-
M9	X	X	-	X
M10	X	X	X	X

Model 1: Line

Fitting the model

```
Z.TRT=as.matrix(TRAIT$treatment-1) #changes treatment from 1,2 to 0,1
Z.Line=as.matrix(model.matrix(~factor(TRAIT$line)-1))

ETA=list(
          trt=list(X=Z.TRT,model='FIXED'),
              line=list(X=Z.Line,model='BRR',saveEffects=T)
        )

# Fitting Model
fmM1=BGLR(y=y,ETA=ETA,saveAt='M1_',nIter=nIter,burnIn=burnIn,thin=thin,verbose=verbose)
YHat=data.frame(ID=TRAIT$line, TRT=TRAIT$treatment,model='M1',yHat=fmM1$yHat)
save(fmM1,YHat,file='fmM1.RData')
```

Posterior Analysis

```
B.TRT=read.table('M1_ETA_trt_b.dat',header=F)[-(1:(burnIn/thin)) ,]
B.Line=readBinMat('M1_ETA_line_b.bin')

vTrt=rep(NA,nrow(B.Line))
vLine=vTrt
vE=vTrt
vModel=vTrt

for(i in 1:nrow(B.Line)){
       yHatTRT=Z.TRT*B.TRT[i]
       vTrt[i]=var(yHatTRT)

       yHatLine=Z.Line%*%B.Line[i,]
       vLine[i]=var(yHatLine)

       yHat=yHatTRT+yHatLine
       vModel[i]=var(yHat)
       eHat=y-yHat
       vE[i]=var(eHat)

}

VAR['M1','TRT']=mean(vTrt)
VAR.LOW['M1','TRT']=quantile(vTrt,prob=.025)
VAR.UP['M1','TRT']=quantile(vTrt,prob=.975)

VAR['M1','Line']=mean(vLine)
VAR.LOW['M1','Line']=quantile(vLine,prob=.025)
VAR.UP['M1','Line']=quantile(vLine,prob=.975)

VAR['M1','Model']=mean(vModel)
VAR.LOW['M1','Model']=quantile(vModel,prob=.025)
VAR.UP['M1','Model']=quantile(vModel,prob=.975)

VAR['M1','Error']=mean(vE)
VAR.LOW['M1','Error']=quantile(vE,prob=.025)
VAR.UP['M1','Error']=quantile(vE,prob=.975)
```

Model 2: Line + SNP

Fitting the model

```
Z.TRT=as.matrix(TRAIT$treatment-1)
Z.Line=as.matrix(model.matrix(~factor(TRAIT$line)-1))
# Extracting all PCs
EVD=eigen(G_SNP)
PC_SNP=EVD$vectors[,EVD$values>1e-5]
for(i in 1:ncol(PC_SNP)){
       PC_SNP[,i]=PC_SNP[,i]*sqrt(EVD$values[i])
}

ETA=list(
          trt=list(X=Z.TRT,model='FIXED'),
              line=list(X=Z.Line,model='BRR',saveEffects=T),
          SNP=list(X=PC_SNP,model='BRR',saveEffects=T)
        )

# Fitting Model
fmM2=BGLR(y=y,ETA=ETA,saveAt='M2_',nIter=nIter,burnIn=burnIn,thin=thin,verbose=verbose)
YHat=data.frame(ID=TRAIT$line, TRT=TRAIT$treatment,model='M2',yHat=fmM2$yHat)
save(fmM2,YHat,file='fmM2.RData')
```

Posterior Analysis

```
B.TRT=read.table('M2_ETA_trt_b.dat',header=F)[-(1:(burnIn/thin)) ,]
B.Line=readBinMat('M2_ETA_line_b.bin')
B.SNP=readBinMat('M2_ETA_SNP_b.bin')

vTrt=rep(NA,nrow(B.Line))
vLine=vTrt
vSNP=vTrt
vE=vTrt
vModel=vTrt

for(i in 1:nrow(B.Line)){
       yHatTRT=Z.TRT*B.TRT[i]
       vTrt[i]=var(yHatTRT)

       yHatLine=Z.Line%*%B.Line[i,]
       vLine[i]=var(yHatLine)

       yHatSNP=PC_SNP%*%B.SNP[i,]
       vSNP[i]=var(yHatSNP)

       yHat=yHatTRT+yHatLine+yHatSNP
       vModel[i]=var(yHat)
       eHat=y-yHat
       vE[i]=var(eHat)

}

VAR['M2','TRT']=mean(vTrt)
VAR.LOW['M2','TRT']=quantile(vTrt,prob=.025)
VAR.UP['M2','TRT']=quantile(vTrt,prob=.975)

VAR['M2','Line']=mean(vLine)
VAR.LOW['M2','Line']=quantile(vLine,prob=.025)
VAR.UP['M2','Line']=quantile(vLine,prob=.975)

VAR['M2','SNP']=mean(vSNP)
VAR.LOW['M2','SNP']=quantile(vSNP,prob=.025)
VAR.UP['M2','SNP']=quantile(vSNP,prob=.975)

VAR['M2','Model']=mean(vModel)
VAR.LOW['M2','Model']=quantile(vModel,prob=.025)
VAR.UP['M2','Model']=quantile(vModel,prob=.975)


VAR['M2','Error']=mean(vE)
VAR.LOW['M2','Error']=quantile(vE,prob=.025)
VAR.UP['M2','Error']=quantile(vE,prob=.975)
```

Model 3: SNP

Fitting the model

```
Z.TRT=as.matrix(TRAIT$treatment-1) #changes treatment from 1,2 to 0,1
EVD=eigen(G_SNP)
# Extracting all PCs
PC_SNP=EVD$vectors[,EVD$values>1e-5]
for(i in 1:ncol(PC_SNP)){
       PC_SNP[,i]=PC_SNP[,i]*sqrt(EVD$values[i])
}

ETA=list(
          trt=list(X=Z.TRT,model='FIXED'),
          SNP=list(X=PC_SNP,model='BRR',saveEffects=T)
        )

# Fitting Model
fmM3=BGLR(y=y,ETA=ETA,saveAt='M3_',nIter=nIter,burnIn=burnIn,thin=thin,verbose=verbose)
YHat=data.frame(ID=TRAIT$line, TRT=TRAIT$treatment,model='M3',yHat=fmM3$yHat)
save(fmM3,YHat,file='fmM3.RData')
```

Posterior Analysis

```
B.TRT=read.table('M3_ETA_trt_b.dat',header=F)[-(1:(burnIn/thin)) ,]
B.SNP=readBinMat('M3_ETA_SNP_b.bin')

vTrt=rep(NA,nrow(B.Line))
vSNP=vTrt
vE=vTrt
vModel=vTrt

for(i in 1:nrow(B.Line)){
       yHatTRT=Z.TRT*B.TRT[i]
       vTrt[i]=var(yHatTRT)

       yHatSNP=PC_SNP%*%B.SNP[i,]
       vSNP[i]=var(yHatSNP)

       yHat=yHatTRT+yHatSNP
       vModel[i]=var(yHat)
       eHat=y-yHat
       vE[i]=var(eHat)

}

VAR['M3','TRT']=mean(vTrt)
VAR.LOW['M3','TRT']=quantile(vTrt,prob=.025)
VAR.UP['M3','TRT']=quantile(vTrt,prob=.975)

VAR['M3','SNP']=mean(vSNP)
VAR.LOW['M3','SNP']=quantile(vSNP,prob=.025)
VAR.UP['M3','SNP']=quantile(vSNP,prob=.975)

VAR['M3','Model']=mean(vModel)
VAR.LOW['M3','Model']=quantile(vModel,prob=.025)
VAR.UP['M3','Model']=quantile(vModel,prob=.975)

VAR['M3','Error']=mean(vE)
VAR.LOW['M3','Error']=quantile(vE,prob=.025)
VAR.UP['M3','Error']=quantile(vE,prob=.975)
```

Model 4: Line + methylation

Fitting the model

```
Z.TRT=as.matrix(TRAIT$treatment-1) #changes treatment from 1,2 to 0,1
Z.Line=as.matrix(model.matrix(~factor(TRAIT$line)-1))
EVD=eigen(G_MET)
# Extracting all PCs
PC_MET=EVD$vectors[,EVD$values>1e-5]
for(i in 1:ncol(PC_MET)){
       PC_MET[,i]=PC_MET[,i]*sqrt(EVD$values[i])
}

ETA=list(
          trt=list(X=Z.TRT,model='FIXED'),
              line=list(X=Z.Line,model='BRR',saveEffects=T),
          MET=list(X=PC_MET,model='BRR',saveEffects=T)
        )

# Fitting Model
fmM4=BGLR(y=y,ETA=ETA,saveAt='M4_',nIter=nIter,burnIn=burnIn,thin=thin,verbose=verbose)
YHat=data.frame(ID=TRAIT$line, TRT=TRAIT$treatment,model='M4',yHat=fmM4$yHat)
save(fmM4,YHat,file='fmM4.RData')
```

Posterior Analysis

```
B.TRT=read.table('M4_ETA_trt_b.dat',header=F)[-(1:(burnIn/thin)) ,]
B.Line=readBinMat('M4_ETA_line_b.bin')
B.MET=readBinMat('M4_ETA_MET_b.bin')

vTrt=rep(NA,nrow(B.Line))
vLine=vTrt
vMET=vTrt
vE=vTrt
vModel=vTrt

for(i in 1:nrow(B.Line)){
       yHatTRT=Z.TRT*B.TRT[i]
       vTrt[i]=var(yHatTRT)

       yHatLine=Z.Line%*%B.Line[i,]
       vLine[i]=var(yHatLine)

       yHatMET=PC_MET%*%B.MET[i,]
       vMET[i]=var(yHatMET)

       yHat=yHatTRT+yHatLine+yHatMET
       vModel[i]=var(yHat)
       eHat=y-yHat
       vE[i]=var(eHat)

}

VAR['M4','TRT']=mean(vTrt)
VAR.LOW['M4','TRT']=quantile(vTrt,prob=.025)
VAR.UP['M4','TRT']=quantile(vTrt,prob=.975)

VAR['M4','Line']=mean(vLine)
VAR.LOW['M4','Line']=quantile(vLine,prob=.025)
VAR.UP['M4','Line']=quantile(vLine,prob=.975)

VAR['M4','MET']=mean(vMET)
VAR.LOW['M4','MET']=quantile(vMET,prob=.025)
VAR.UP['M4','MET']=quantile(vMET,prob=.975)

VAR['M4','Model']=mean(vModel)
VAR.LOW['M4','Model']=quantile(vModel,prob=.025)
VAR.UP['M4','Model']=quantile(vModel,prob=.975)

VAR['M4','Error']=mean(vE)
VAR.LOW['M4','Error']=quantile(vE,prob=.025)
VAR.UP['M4','Error']=quantile(vE,prob=.975)
```

Model 5: MET

Fitting the model

```
Z.TRT=as.matrix(TRAIT$treatment-1) #changes treatment from 1,2 to 0,1
EVD=eigen(G_MET)
# Extracting all PCs
PC_MET=EVD$vectors[,EVD$values>1e-5]
for(i in 1:ncol(PC_MET)){
       PC_MET[,i]=PC_MET[,i]*sqrt(EVD$values[i])
}

ETA=list(
          trt=list(X=Z.TRT,model='FIXED'),
          MET=list(X=PC_MET,model='BRR',saveEffects=T)
        )

# Fitting Model
fmM5=BGLR(y=y,ETA=ETA,saveAt='M5_',nIter=nIter,burnIn=burnIn,thin=thin,verbose=verbose)
YHat=data.frame(ID=TRAIT$line, TRT=TRAIT$treatment,model='M5',yHat=fmM5$yHat)
save(fmM5,YHat,file='fmM5.RData')
```

Posterior Analysis

```
B.TRT=read.table('M5_ETA_trt_b.dat',header=F)[-(1:(burnIn/thin)) ,]
B.MET=readBinMat('M5_ETA_MET_b.bin')

vTrt=rep(NA,nrow(B.Line))
vMET=vTrt
vE=vTrt
vModel=vTrt

for(i in 1:nrow(B.Line)){
       yHatTRT=Z.TRT*B.TRT[i]
       vTrt[i]=var(yHatTRT)

       yHatMET=PC_MET%*%B.MET[i,]
       vMET[i]=var(yHatMET)

       yHat=yHatTRT+yHatMET
       vModel[i]=var(yHat)
       eHat=y-yHat
       vE[i]=var(eHat)

}

VAR['M5','TRT']=mean(vTrt)
VAR.LOW['M5','TRT']=quantile(vTrt,prob=.025)
VAR.UP['M5','TRT']=quantile(vTrt,prob=.975)

VAR['M5','MET']=mean(vMET)
VAR.LOW['M5','MET']=quantile(vMET,prob=.025)
VAR.UP['M5','MET']=quantile(vMET,prob=.975)

VAR['M5','Model']=mean(vModel)
VAR.LOW['M5','Model']=quantile(vModel,prob=.025)
VAR.UP['M5','Model']=quantile(vModel,prob=.975)

VAR['M5','Error']=mean(vE)
VAR.LOW['M5','Error']=quantile(vE,prob=.025)
VAR.UP['M5','Error']=quantile(vE,prob=.975)
```

Model 6: Line + transcript expression

Fitting the model

```
Z.TRT=as.matrix(TRAIT$treatment-1)
Z.Line=as.matrix(model.matrix(~factor(TRAIT$line)-1))
EVD=eigen(G_DE)
# Extracting all PCs
PC_DE=EVD$vectors[,EVD$values>1e-5]
for(i in 1:ncol(PC_DE)){
       PC_DE[,i]=PC_DE[,i]*sqrt(EVD$values[i])
}

ETA=list(
          trt=list(X=Z.TRT,model='FIXED'),
              line=list(X=Z.Line,model='BRR',saveEffects=T),
          DE=list(X=PC_DE,model='BRR',saveEffects=T)
        )

# Fitting Model
fmM6=BGLR(y=y,ETA=ETA,saveAt='M6_',nIter=nIter,burnIn=burnIn,thin=thin,verbose=verbose)
YHat=data.frame(ID=TRAIT$line, TRT=TRAIT$treatment,model='M6',yHat=fmM6$yHat)
save(fmM6,YHat,file='fmM6.RData')
```

Posterior Analysis

```
B.TRT=read.table('M6_ETA_trt_b.dat',header=F)[-(1:(burnIn/thin)) ,]
B.Line=readBinMat('M6_ETA_line_b.bin')
B.DE=readBinMat('M6_ETA_DE_b.bin')

vTrt=rep(NA,nrow(B.Line))
vLine=vTrt
vDE=vTrt
vE=vTrt
vModel=vTrt

for(i in 1:nrow(B.Line)){
       yHatTRT=Z.TRT*B.TRT[i]
       vTrt[i]=var(yHatTRT)

       yHatLine=Z.Line%*%B.Line[i,]
       vLine[i]=var(yHatLine)

       yHatDE=PC_DE%*%B.DE[i,]
       vDE[i]=var(yHatDE)

       yHat=yHatTRT+yHatLine+yHatDE
       vModel[i]=var(yHat)
       eHat=y-yHat
       vE[i]=var(eHat)

}

VAR['M6','TRT']=mean(vTrt)
VAR.LOW['M6','TRT']=quantile(vTrt,prob=.025)
VAR.UP['M6','TRT']=quantile(vTrt,prob=.975)

VAR['M6','Line']=mean(vLine)
VAR.LOW['M6','Line']=quantile(vLine,prob=.025)
VAR.UP['M6','Line']=quantile(vLine,prob=.975)

VAR['M6','DE']=mean(vDE)
VAR.LOW['M6','DE']=quantile(vDE,prob=.025)
VAR.UP['M6','DE']=quantile(vDE,prob=.975)

VAR['M6','Model']=mean(vModel)
VAR.LOW['M6','Model']=quantile(vModel,prob=.025)
VAR.UP['M6','Model']=quantile(vModel,prob=.975)

VAR['M6','Error']=mean(vE)
VAR.LOW['M6','Error']=quantile(vE,prob=.025)
VAR.UP['M6','Error']=quantile(vE,prob=.975)
```

Model 7: Transcript expression

Fitting the model

```
Z.TRT=as.matrix(TRAIT$treatment-1)
EVD=eigen(G_DE)
# Extracting all PCs
PC_DE=EVD$vectors[,EVD$values>1e-5]
for(i in 1:ncol(PC_DE)){
       PC_DE[,i]=PC_DE[,i]*sqrt(EVD$values[i])
}

ETA=list(
          trt=list(X=Z.TRT,model='FIXED'),
          DE=list(X=PC_DE,model='BRR',saveEffects=T)
        )

# Fitting Model
fmM7=BGLR(y=y,ETA=ETA,saveAt='M7_',nIter=nIter,burnIn=burnIn,thin=thin,verbose=verbose)
YHat=data.frame(ID=TRAIT$line, TRT=TRAIT$treatment,model='M7',yHat=fmM7$yHat)
save(fmM7,YHat,file='fmM7.RData')
```

Posterior Analysis

```
B.TRT=read.table('M7_ETA_trt_b.dat',header=F)[-(1:(burnIn/thin)) ,]
B.DE=readBinMat('M7_ETA_DE_b.bin')

vTrt=rep(NA,nrow(B.Line))
vDE=vTrt
vE=vTrt
vModel=vTrt

for(i in 1:nrow(B.Line)){
       yHatTRT=Z.TRT*B.TRT[i]
       vTrt[i]=var(yHatTRT)

       yHatDE=PC_DE%*%B.DE[i,]
       vDE[i]=var(yHatDE)

       yHat=yHatTRT+yHatDE
       vModel[i]=var(yHat)
       eHat=y-yHat
       vE[i]=var(eHat)

}

VAR['M7','TRT']=mean(vTrt)
VAR.LOW['M7','TRT']=quantile(vTrt,prob=.025)
VAR.UP['M7','TRT']=quantile(vTrt,prob=.975)

VAR['M7','DE']=mean(vDE)
VAR.LOW['M7','DE']=quantile(vDE,prob=.025)
VAR.UP['M7','DE']=quantile(vDE,prob=.975)

VAR['M7','Model']=mean(vModel)
VAR.LOW['M7','Model']=quantile(vModel,prob=.025)
VAR.UP['M7','Model']=quantile(vModel,prob=.975)

VAR['M7','Error']=mean(vE)
VAR.LOW['M7','Error']=quantile(vE,prob=.025)
VAR.UP['M7','Error']=quantile(vE,prob=.975)
```

Model 8: Line + SNP + methylation

Fitting the model

```
Z.TRT=as.matrix(TRAIT$treatment-1)
Z.Line=as.matrix(model.matrix(~factor(TRAIT$line)-1))

ETA=list(
          trt=list(X=Z.TRT,model='FIXED'),
              line=list(X=Z.Line,model='BRR',saveEffects=T),
          SNP=list(X=PC_SNP,model='BRR',saveEffects=T),
          MET=list(X=PC_MET,model='BRR',saveEffects=T)
        )

# Fitting Model
fmM8=BGLR(y=y,ETA=ETA,saveAt='M8_',nIter=nIter,burnIn=burnIn,thin=thin,verbose=verbose)
YHat=data.frame(ID=TRAIT$line, TRT=TRAIT$treatment,model='M8',yHat=fmM8$yHat)
save(fmM8,YHat,file='fmM8.RData')
```

Posterior Analysis

```
B.TRT=read.table('M8_ETA_trt_b.dat',header=F)[-(1:(burnIn/thin)) ,]
B.Line=readBinMat('M8_ETA_line_b.bin')
B.SNP=readBinMat('M8_ETA_SNP_b.bin')
B.MET=readBinMat('M8_ETA_MET_b.bin')

vTrt=rep(NA,nrow(B.Line))
vLine=vTrt
vSNP=vTrt
vMET=vTrt
vE=vTrt
vModel=vTrt

for(i in 1:nrow(B.Line)){
       yHatTRT=Z.TRT*B.TRT[i]
       vTrt[i]=var(yHatTRT)

       yHatLine=Z.Line%*%B.Line[i,]
       vLine[i]=var(yHatLine)

       yHatSNP=PC_SNP%*%B.SNP[i,]
       vSNP[i]=var(yHatSNP)

       yHatMET=PC_MET%*%B.MET[i,]
       vMET[i]=var(yHatMET)

       yHat=yHatTRT+yHatLine+yHatSNP+yHatMET
       vModel[i]=var(yHat)
       eHat=y-yHat
       vE[i]=var(eHat)

}

VAR['M8','TRT']=mean(vTrt)
VAR.LOW['M8','TRT']=quantile(vTrt,prob=.025)
VAR.UP['M8','TRT']=quantile(vTrt,prob=.975)

VAR['M8','Line']=mean(vLine)
VAR.LOW['M8','Line']=quantile(vLine,prob=.025)
VAR.UP['M8','Line']=quantile(vLine,prob=.975)

VAR['M8','SNP']=mean(vSNP)
VAR.LOW['M8','SNP']=quantile(vSNP,prob=.025)
VAR.UP['M8','SNP']=quantile(vSNP,prob=.975)

VAR['M8','MET']=mean(vMET)
VAR.LOW['M8','MET']=quantile(vMET,prob=.025)
VAR.UP['M8','MET']=quantile(vMET,prob=.975)

VAR['M8','Model']=mean(vModel)
VAR.LOW['M8','Model']=quantile(vModel,prob=.025)
VAR.UP['M8','Model']=quantile(vModel,prob=.975)

VAR['M8','Error']=mean(vE)
VAR.LOW['M8','Error']=quantile(vE,prob=.025)
VAR.UP['M8','Error']=quantile(vE,prob=.975)
```

Model 9: Line + SNP + transcript expression

Fitting the model

```
Z.TRT=as.matrix(TRAIT$treatment-1)
Z.Line=as.matrix(model.matrix(~factor(TRAIT$line)-1))

ETA=list(
          trt=list(X=Z.TRT,model='FIXED'),
              line=list(X=Z.Line,model='BRR',saveEffects=T),
          SNP=list(X=PC_SNP,model='BRR',saveEffects=T),
          DE=list(X=PC_DE,model='BRR',saveEffects=T)
        )

# Fitting Model
fmM9=BGLR(y=y,ETA=ETA,saveAt='M9_',nIter=nIter,burnIn=burnIn,thin=thin,verbose=verbose)
YHat=data.frame(ID=TRAIT$line, TRT=TRAIT$treatment,model='M9',yHat=fmM9$yHat)
save(fmM9,YHat,file='fmM9.RData')
```

Posterior analyses

```
B.TRT=read.table('M9_ETA_trt_b.dat',header=F)[-(1:(burnIn/thin)) ,]
B.Line=readBinMat('M9_ETA_line_b.bin')
B.SNP=readBinMat('M9_ETA_SNP_b.bin')
B.DE=readBinMat('M9_ETA_DE_b.bin')

vTrt=rep(NA,nrow(B.Line))
vLine=vTrt
vSNP=vTrt
vDE=vTrt
vE=vTrt
vModel=vTrt

for(i in 1:nrow(B.Line)){
       yHatTRT=Z.TRT*B.TRT[i]
       vTrt[i]=var(yHatTRT)

       yHatLine=Z.Line%*%B.Line[i,]
       vLine[i]=var(yHatLine)

       yHatSNP=PC_SNP%*%B.SNP[i,]
       vSNP[i]=var(yHatSNP)

       yHatDE=PC_DE%*%B.DE[i,]
       vDE[i]=var(yHatDE)

       yHat=yHatTRT+yHatLine+yHatSNP+yHatDE
       vModel[i]=var(yHat)
       eHat=y-yHat
       vE[i]=var(eHat)

}

VAR['M9','TRT']=mean(vTrt)
VAR.LOW['M9','TRT']=quantile(vTrt,prob=.025)
VAR.UP['M9','TRT']=quantile(vTrt,prob=.975)

VAR['M9','Line']=mean(vLine)
VAR.LOW['M9','Line']=quantile(vLine,prob=.025)
VAR.UP['M9','Line']=quantile(vLine,prob=.975)

VAR['M9','SNP']=mean(vSNP)
VAR.LOW['M9','SNP']=quantile(vSNP,prob=.025)
VAR.UP['M9','SNP']=quantile(vSNP,prob=.975)

VAR['M9','DE']=mean(vDE)
VAR.LOW['M9','DE']=quantile(vDE,prob=.025)
VAR.UP['M9','DE']=quantile(vDE,prob=.975)

VAR['M9','Model']=mean(vModel)
VAR.LOW['M9','Model']=quantile(vModel,prob=.025)
VAR.UP['M9','Model']=quantile(vModel,prob=.975)

VAR['M9','Error']=mean(vE)
VAR.LOW['M9','Error']=quantile(vE,prob=.025)
VAR.UP['M9','Error']=quantile(vE,prob=.975)
```

Model 10: Line + SNP + methyaltion + transcript expression

Fitting the model

```
Z.TRT=as.matrix(TRAIT$treatment-1)
Z.Line=as.matrix(model.matrix(~factor(TRAIT$line)-1))

ETA=list(
          trt=list(X=Z.TRT,model='FIXED'),
              line=list(X=Z.Line,model='BRR',saveEffects=T),
          SNP=list(X=PC_SNP,model='BRR',saveEffects=T),
              MET=list(X=PC_MET,model='BRR',saveEffects=T) ,
          DE=list(X=PC_DE,model='BRR',saveEffects=T)
        )

# Fitting Model
fmM10=BGLR(y=y,ETA=ETA,saveAt='M10_',nIter=nIter,burnIn=burnIn,thin=thin,verbose=verbose)
YHat=data.frame(ID=TRAIT$line, TRT=TRAIT$treatment,model='M10',yHat=fmM10$yHat)
save(fmM10,YHat,file='fmM10.RData')
```

Posterior analyses

```
B.TRT=read.table('M10_ETA_trt_b.dat',header=F)[-(1:(burnIn/thin)) ,]
B.Line=readBinMat('M10_ETA_line_b.bin')
B.SNP=readBinMat('M10_ETA_SNP_b.bin')
B.MET=readBinMat('M10_ETA_MET_b.bin')
B.DE=readBinMat('M10_ETA_DE_b.bin')

vTrt=rep(NA,nrow(B.SNP))
vLine=vTrt
vSNP=vTrt
vMET=vTrt
vDE=vTrt
vE=vTrt
vModel=vTrt

for(i in 1:nrow(B.Line)){
       yHatTRT=Z.TRT*B.TRT[i]
       vTrt[i]=var(yHatTRT)

       yHatLine=Z.Line%*%B.Line[i,]
       vLine[i]=var(yHatLine)

       yHatSNP=PC_SNP%*%B.SNP[i,]
       vSNP[i]=var(yHatSNP)

       yHatMET=PC_MET%*%B.MET[i,]
       vMET[i]=var(yHatMET)

       yHatDE=PC_DE%*%B.DE[i,]
       vDE[i]=var(yHatDE)

       yHat=yHatTRT+yHatLine+yHatSNP+yHatMET+yHatDE
       vModel[i]=var(yHat)
       eHat=y-yHat
       vE[i]=var(eHat)

}


VAR['M10','TRT']=mean(vTrt)
VAR.LOW['M10','TRT']=quantile(vTrt,prob=.025)
VAR.UP['M10','TRT']=quantile(vTrt,prob=.975)

VAR['M10','Line']=mean(vLine)
VAR.LOW['M10','Line']=quantile(vLine,prob=.025)
VAR.UP['M10','Line']=quantile(vLine,prob=.975)

VAR['M10','SNP']=mean(vSNP)
VAR.LOW['M10','SNP']=quantile(vSNP,prob=.025)
VAR.UP['M10','SNP']=quantile(vSNP,prob=.975)

VAR['M10','MET']=mean(vMET)
VAR.LOW['M10','MET']=quantile(vMET,prob=.025)
VAR.UP['M10','MET']=quantile(vMET,prob=.975)

VAR['M10','DE']=mean(vDE)
VAR.LOW['M10','DE']=quantile(vDE,prob=.025)
VAR.UP['M10','DE']=quantile(vDE,prob=.975)
```

```
VAR['M10','Model']=mean(vModel)
VAR.LOW['M10','Model']=quantile(vModel,prob=.025)
VAR.UP['M10','Model']=quantile(vModel,prob=.975)

VAR['M10','Error']=mean(vE)
VAR.LOW['M10','Error']=quantile(vE,prob=.025)
VAR.UP['M10','Error']=quantile(vE,prob=.975)
```

Saving the complete explained variance table

```
write.table(VAR,file='VAR.dat')
write.table(VAR.LOW,file='VAR_LOW.dat')
write.table(VAR.UP,file='VAR_UP.dat')
```

Example of the VAR.dat table with the trait nitrogen_uptake_nir

```
           TRT      Line       SNP       MET        DE     Model     Error
M1  0.06592495 0.5305539        NA        NA        NA 0.5975973 0.3999818
M2  0.06357194 0.3032075 0.2442125        NA        NA 0.6062930 0.3992894
M3  0.06260539        NA 0.4818488        NA        NA 0.5486374 0.4460318
M4  0.06491917 0.4715714        NA 0.1563975        NA 0.7051273 0.3059883
M5  0.06824661        NA        NA 0.3641272        NA 0.4297179 0.5806501
M6  0.04284406 0.3896354        NA        NA 0.2465880 0.7367506 0.2673206
M7  0.03589124        NA        NA        NA 0.5957626 0.6449104 0.3538081
M8  0.06252977 0.2687204 0.2252276 0.1296465        NA 0.6937825 0.3176982
M9  0.04648366 0.2377194 0.2075651        NA 0.1960835 0.7262172 0.2816113
M10 0.04571742 0.2228169 0.1731009 0.1013929 0.1601447 0.7534889 0.2612752
```
