# Supplementary material for: Integration of DNA Methylation and Transcriptome Data Improves Complex Trait Prediction in Hordeum vulgare
Source: Plants (Basel). 2022 Aug 24;11(17):2190. doi: 10.3390/plants11172190 (PMC9459846; doi:10.3390/plants11172190)
Supplement: Supplementary file 1 [file plants-11-02190-s001.zip › File S2.pdf]

Cross-validation

Pernille Bjarup Hansen and Gustavo de los Campos

8/9/2018

Parameters

```
 maxPropNA=0.05
 maxPropZeros=0.9
 maxPropOnes=1
 trait="nitrogen_uptake_nir" # 'grain_yield'  'protein'   'tkw' 'nitrogen_uptake_nir'
 nIter=12000
 burnIn=2000
 thin=2
 verbose=F #T/F
 cvID=1
##
```

Loading the data

```
path='/mnt/research/quantgen/projects/pernille'
setwd(paste(path,'/TEST/output',sep=''))
library(BGLR);library(BGData)
load('../../PREPARATION/output/PREP.RData')
y=scale(TRAIT[,trait])
```

Folders

```
dir.create(paste0('CV'))
setwd(paste0('CV'))
dir.create(trait)
setwd(trait)
```

Setting seed

```
set.seed(195021)
seeds=sample(1000:5000,size=1000)
set.seed(seeds[cvID])
```

Creating folds

Creating folds with genotypic replicates within same fold (CV1)

```
ID<- data.frame(ID=seq(1,75,1),fold=rep(0,75))

for (i in 2:5){
 s <- sample(ID[ID[,2]==0,1],size=15,replace=FALSE)
 ID[s,2]<-i
 }

ID[ID[,2]==0,2]<-1

id <- rep(ID[,1], each=2)
fold <- rep(ID[,2], each=2)
idfold <-cbind(id,fold)
idfold <- idfold[-c(52,65,113,125),]

t <- TRAIT[order(TRAIT[,"line"]),]
t1<-cbind(t,idfold[,2])
names(t1)[7] <-'folds'

t2 <- t1[order(t1$treatment),]
names(t2)[7] <- 'CV1'

#Get the data set back to the original order
row.order <- rownames(TRAIT)
t2 <- t2[row.order,]

#folds
CV1 <- t2$CV1
```

Preparing predictors

```
Z.TRT=as.matrix(TRAIT$treatment-1) #changes treatment from 1,2 to 0,1
Z.Line=as.matrix(model.matrix(~factor(TRAIT$line)-1))

EVD=eigen(G_SNP)
# Extracting all PCs
PC_SNP=EVD$vectors[,EVD$values>1e-5]
for(i in 1:ncol(PC_SNP)){
       PC_SNP[,i]=PC_SNP[,i]*sqrt(EVD$values[i])
}

EVD=eigen(G_MET)
# Extracting all PCs
PC_MET=EVD$vectors[,EVD$values>1e-5]
for(i in 1:ncol(PC_MET)){
       PC_MET[,i]=PC_MET[,i]*sqrt(EVD$values[i])
}

EVD=eigen(G_DE)
# Extracting all PCs
PC_DE=EVD$vectors[,EVD$values>1e-5]
for(i in 1:ncol(PC_DE)){
       PC_DE[,i]=PC_DE[,i]*sqrt(EVD$values[i])
}

ETA=list(
               trt=list(X=Z.TRT,model='FIXED'),
               line=list(X=Z.Line,model='BRR',saveEffects=F),
               SNP=list(X=PC_SNP,model='BRR',saveEffects=F),
               MET=list(X=PC_MET,model='BRR',saveEffects=F),
               DE=list(X=PC_DE,model='BRR',saveEffects=F)

       )
```

Run cross-validation on CV1 folds

```
YHatCV=matrix(nrow=length(y),ncol=11,NA)
colnames(YHatCV)=c('TRT','M1','M2','M3', 'M4','M5','M6','M7','M8','M9','M10')


for(i in 1:5){
                print(i)
                yNA=y
                tst<-CV1==i
                yNA[tst]=NA

                # TRT
                  fm=BGLR(y=yNA,ETA=ETA['trt'],nIter=nIter,burnIn=burnIn,thin=thin,verbose=ve
rbose)
                  YHatCV[tst,'TRT']=fm$yHat[tst]

                # TRT+LINE
                  fm=BGLR(y=yNA,ETA=ETA[c('trt','line')],nIter=nIter,burnIn=burnIn,thin=thin,
verbose=verbose)
                  YHatCV[tst,'M1']=fm$yHat[tst]

                # TRT+LINE+SNP
                  fm=BGLR(y=yNA,ETA=ETA[c('trt','line','SNP')],nIter=nIter,burnIn=burnIn,thin
=thin, verbose=verbose)
                  YHatCV[tst,'M2']=fm$yHat[tst]

                # TRT+SNP
                  fm=BGLR(y=yNA,ETA=ETA[c('trt','line','SNP')],nIter=nIter,burnIn=burnIn,thin
=thin, verbose=verbose)
                  YHatCV[tst,'M3']=fm$yHat[tst]

                # TRT+LINE+MET
                  fm=BGLR(y=yNA,ETA=ETA[c('trt','SNP')],nIter=nIter,burnIn=burnIn,thin=thin,
 verbose=verbose)
                  YHatCV[tst,'M4']=fm$yHat[tst]

                # TRT+MET
                  fm=BGLR(y=yNA,ETA=ETA[c('trt','MET')],nIter=nIter,burnIn=burnIn,thin=thin,
 verbose=verbose)
                  YHatCV[tst,'M5']=fm$yHat[tst]

                # TRT+LINE+DE
                  fm=BGLR(y=yNA,ETA=ETA[c('trt','line','DE')],nIter=nIter,burnIn=burnIn,thin=
thin, verbose=verbose)
                  YHatCV[tst,'M6']=fm$yHat[tst]

                # TRT+DE
                  fm=BGLR(y=yNA,ETA=ETA[c('trt','DE')],nIter=nIter,burnIn=burnIn,thin=thin, v
erbose=verbose)
                  YHatCV[tst,'M7']=fm$yHat[tst]

                # TRT+LINE+SNP+MET
                  fm=BGLR(y=yNA,ETA=ETA[c('trt','line','SNP', 'MET')],nIter=nIter,burnIn=burn
In, thin=thin, verbose=verbose)
                  YHatCV[tst,'M8']=fm$yHat[tst]

                 # TRT+LINE+SNP+DE
                  fm=BGLR(y=yNA,ETA=ETA[c('trt','line','SNP','DE')],nIter=nIter,burnIn=burnI
```

```
n, thin=thin, verbose=verbose)
                  YHatCV[tst,'M9']=fm$yHat[tst]

                # TRT+LINE+SNP+MET+DE
                  fm=BGLR(y=yNA,ETA=ETA[c('trt','line','SNP','DE', 'MET')],nIter=nIter,burnIn
=burnIn, thin=thin, verbose=verbose)
                  YHatCV[tst,'M10']=fm$yHat[tst]
}
unlink('*.dat')

save(y,YHatCV,t2, CV1, file=paste0('PRED1_',cvID,'.RData'))
```

Correlation

The correlation, as a measure of accuracy, between the predicted (YHat) and observed (y) can easily be investigated from the output

```
#Evaluation of CV1
load('PRED1_1.RData')
cor(YHatCV,y)

#Evaluation of CV1 within conditions
set <- t2$treatment==1
cor(YHatCV[set,],y[set,])
set <- t2$treatment==2
cor(YHatCV[set,],y[set,])
```
