# Supplementary material for: Integration of DNA Methylation and Transcriptome Data Improves Complex Trait Prediction in Hordeum vulgare
Source: Plants (Basel). 2022 Aug 24;11(17):2190. doi: 10.3390/plants11172190 (PMC9459846; doi:10.3390/plants11172190)
Supplement: Supplementary file 1 [file plants-11-02190-s001.zip › plants-1760595 R5 supplementary.pdf]

Table S1. Parameters included in the linear mixed model regression in the mediation analysis.

Path	Response	Fixed effect	Random effect
C	Trait	Treatment	Line
A_1	Methylomic	Treatment	Line
A_2	Transcriptomic	Treatment	Line
B_1	Trait	Treatment + methylomic	Line
B_2	Trait	Treatment + transcriptomic	Line

Table S2. Parameters included in the Bayesian prediction models and their model abbreviations.

Model	Treatment	Line	SNPs	DNA methylation	Gene expression
M1	X	X			
M2	X	X	X		
M3	X	X		X	
M4	X	X			X
M5	X	X	X	X	
M6	X	X	X		X
M7	x	X	X	X	X

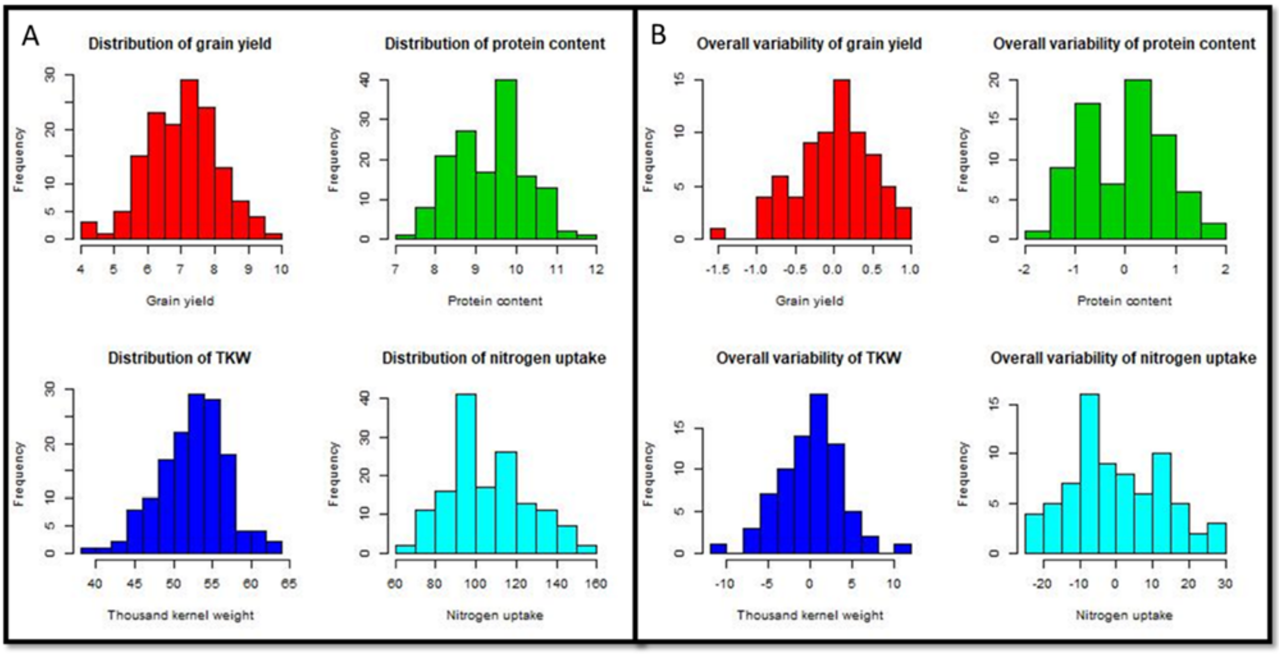

Figure S1. A. Normal distribution of the traits. B. Distribution of the overall variabilities (distribution of random effects) of the traits. For all traits, the normality and variability were investigated using a linear mixed model approach using the "lme4" package [73] in R [74].

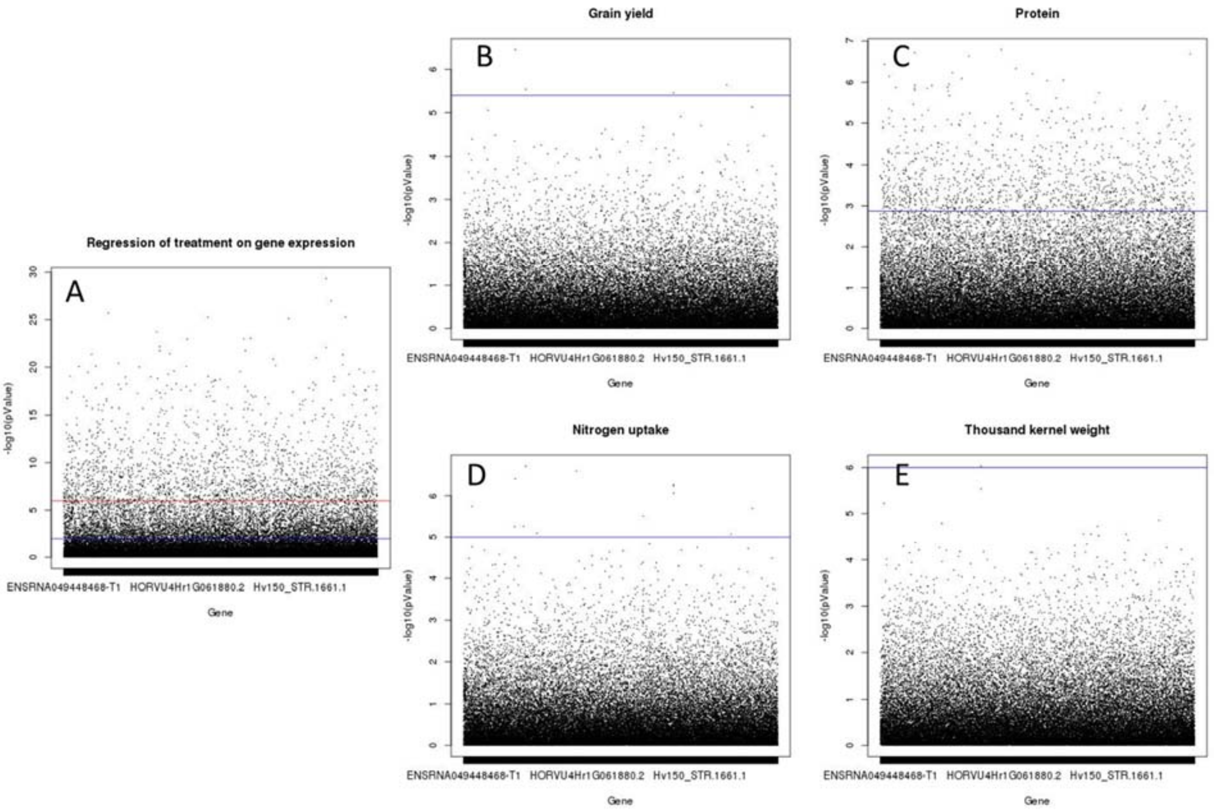

Figure S2. Step two and three in the mediation analysis using the transcriptomic data set. A. shows the regression of treatment on gene expression. The blue line represents the significance level after adjusting for FDR with BH and the red line after adjusting with Bonferroni. B-E shows the regression of treatment and gene expression on the traits. The blue line shows the significance level after adjusting for FDR using BH.

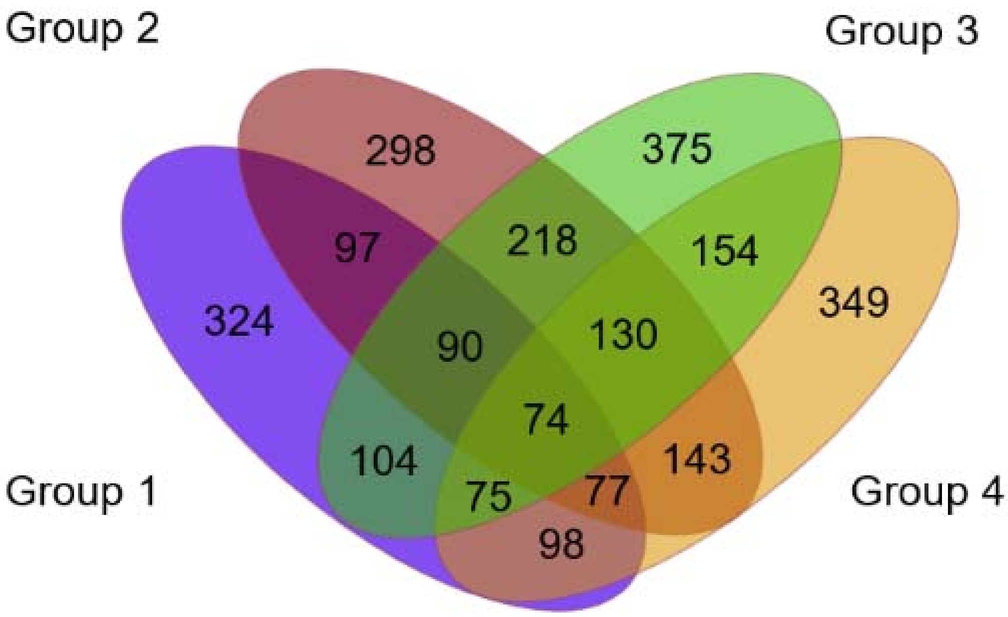

Figure S3. Seventy-four genes were differentially expressed in all groups. Graph made with 'Inkscape' [86].

Table S3. Pfam domains in the 74 de genes

PF00854	POT family
PF02183	Homeobox associated leucine zipper
PF00046	Homeobox domain
PF14543	Xylanase inhibitor N-terminal
PF14541	Xylanase inhibitor C-terminal
PF01161	Phosphatidylethanolamine-binding protein
PF08596	Lethal giant larvae(Lgl) like, C-terminal
PF00117	Glutamine amidotransferase class-I
PF06418	CTP synthase N-terminus
PF00012	Hsp70 protein
PF10604	Polyketide cyclase / dehydrase and lipid transport
PF00481	Protein phosphatase 2C
PF03107	C1 domain
PF13905	Thioredoxin-like
PF13905	Thioredoxin-like
PF10604	Polyketide cyclase / dehydrase and lipid transport
PF00494	Squalene/phytoene synthase
PF00514	Armadillo/beta-catenin-like repeat
PF04564	U-box domain
PF03055	Retinal pigment epithelial membrane protein
PF07714	Protein tyrosine kinase
PF00010	Helix-loop-helix DNA-binding domain
PF13912	C2H2-type zinc finger
PF13912	C2H2-type zinc finger
PF00447	HSF-type DNA-binding
PF13963	Transposase-associated domain
PF00257	Dehydrin
PF01373	Glycosyl hydrolase family 14
PF00847	AP2 domain
PF03055	Retinal pigment epithelial membrane protein
PF13963	Transposase-associated domain
PF13963	Transposase-associated domain
PF13963	Transposase-associated domain
PF03055	Retinal pigment epithelial membrane protein

PF13963	Transposase-associated domain
PF13912	C2H2-type zinc finger
PF13912	C2H2-type zinc finger
PF03055	Retinal pigment epithelial membrane protein
PF10839	Protein of unknown function (DUF2647)
PF00847	AP2 domain
PF00249	Myb-like DNA-binding domain
PF02788	Ribulose bisphosphate carboxylase large chain, N-terminal domain
PF00016	Ribulose bisphosphate carboxylase large chain, catalytic domain
PF00249	Myb-like DNA-binding domain
PF00249	Myb-like DNA-binding domain
PF00249	Myb-like DNA-binding domain
PF00249	Myb-like DNA-binding domain
PF03619	Organic solute transporter Ostalpha
PF13639	Ring finger domain
PF00046	Homeobox domain
PF02183	Homeobox associated leucine zipper
PF00847	AP2 domain
PF05553	Cotton fibre expressed protein
PF14364	Domain of unknown function (DUF4408)
PF10839	Protein of unknown function (DUF2647)

Table S4. Variance components estimates and prediction accuracy by model.

Grain yield g m^{-2}	Proportion of variance explained (97.5 % posterior confidence region): Predictors							Cross-validation prediction correlation (SD): CV	
	Treatment	Line	SNPs	Methylation	Gene expression	Model	Error	WW[b]	WS[b]
M1	0.15 (0.07;0.25)	0.35 (0.18;0.53)				0.50 (0.31;0.70)	0.51 (0.41;0.65)	0.00 (0.28)	0.00 (0.27)
M2	0.14 (0.06;0.24)	0.17 (0.07;0.31)	0.24 (0.11;0.44)			0.55 (0.39;0.74)	0.48 (0.39;0.58)	0.23 (0.28)	0.16 (0.30)
M3	0.14 (0.06;0.25)	0.29 (0.13;0.47)		0.18 (0.07;0.36)		0.62 (0.41;0.84)	0.40 (0.22;0.58)	0.00 (0.25)	-0.01 (0.26)
M4	0.10 (0.02;0.22)	0.25 (0.11;0.43)			0.28 (0.11;0.51)	0.68 (0.46;0.88)	0.32 (0.18;0.50)	0.14 (0.25)	0.46 (0.21)
M5	0.14 (0.06;0.24)	0.14 (0.05;0.29)	0.22 (0.09;0.40)	0.14 (0.06;0.29)		0.65 (0.47;0.85)	0.38 (0.23;0.52)	0.23 (0.28)	0.15 (0.30)
M6	0.12 (0.03;0.24)	0.14 (0.05;0.27)	0.21 (0.08;0.39)		0.19 (0.07;0.37)	0.68 (0.49;0.88)	0.34 (0.20;0.49)	0.22 (0.26)	0.30 (0.27)

M7	0.12 (0.03;0.24)	0.13 (0.05;0.27)	0.19 (0.07;0.37)	0.11 (0.04;0.22)	0.15 (0.06;0.31)	0.72 (0.54;0.90)	0.31 (0.18;0.46)	0.23 (0.27)	0.28 (0.28)
Protein (% of dry matter)									
M1	0.01 (0.00;0.03)	0.85 (0.73;0.97)				0.86 (0.74;0.98)	0.14 (0.10;0.18)	0.00 (0.28)	0.00 (0.27)
M2	0.01 (0.00;0.03)	0.57 (0.30;0.84)	0.28 (0.08;0.54)			0.86 (0.73;0.99)	0.14 (0.10;0.18)	0.13 (0.23)	0.13 (0.24)
M3	0.01 (0.00;0.03)	0.80 (0.65;0.95)		0.07 (0.04;0.12)		0.90 (0.77;1.02)	0.12 (0.08;0.17)	0.04 (0.26)	0.03 (0.27)
M4	0.02 (0.00;0.05)	0.73 (0.57;0.90)			0.10 (0.05;0.17)	0.90 (0.78;1.02)	0.11 (0.07;0.16)	0.45 (0.22)	0.30 (0.23)
M5	0.01 (0.00;0.03)	0.56 (0.24;0.82)	0.24 (0.07;0.54)	0.06 (0.04;0.11)		0.89 (0.78;1.01)	0.12 (0.07;0.18)	0.14 (0.23)	0.13 (0.24)
M6	0.01 (0.00;0.04)	0.51 (0.24;0.75)	0.23 (0.07;0.52)		0.09 (0.04;0.15)	0.90 (0.78;1.01)	0.11 (0.07;0.16)	0.36 (0.21)	0.22 (0.24)
M7	0.02 (0.00;0.05)	0.48 (0.20;0.73)	0.22 (0.06;0.52)	0.05 (0.03;0.09)	0.08 (0.04;0.16)	0.91 (0.79;1.03)	0.12 (0.07;0.17)	0.37 (0.22)	0.24 (0.24)

TKW (g)									
M1	0.12 (0.07;0.18)	0.71 (0.58;0.84)				0.83 (0.69;0.97)	0.17 (0.13;0.22)	0.00 (0.27)	0.00 (0.27)
M2	0.12 (0.07;0.17)	0.26 (0.09;0.51)	0.46 (0.19;0.72)			0.83 (0.71;0.97)	0.16 (0.13;0.22)	0.35 (0.22)	0.27 (0.27)
M3	0.13 (0.07;0.19)	0.67 (0.53;0.82)		0.08 (0.04;0.13)		0.87 (0.74;1.00)	0.14 (0.09;0.21)	0.02 (0.27)	0.01 (0.27)
M4	0.10 (0.04;0.17)	0.64 (0.48;0.80)			0.10 (0.05;0.18)	0.88 (0.74;1.00)	0.13 (0.09;0.20)	0.27 (0.24)	0.21 (0.26)
M5	0.13 (0.07;0.19)	0.23 (0.07;0.48)	0.44 (0.18;0.70)	0.07 (0.04;0.12)		0.87 (0.74;1.00)	0.14 (0.10;0.21)	0.36 (0.22)	0.27 (0.27)
M6	0.11 (0.05;0.18)	0.24 (0.08;0.49)	0.43 (0.16;0.69)		0.08 (0.04;0.14)	0.87 (0.75;1.00)	0.14 (0.09;0.20)	0.37 (0.21)	0.28 (0.27)
M7	0.11 (0.05;0.19)	0.23 (0.06;0.50)	0.40 (0.11;0.69)	0.06 (0.03;0.10)	0.07 (0.03;0.13)	0.89 (0.76;1.01)	0.14 (0.09;0.20)	0.37 (0.21)	0.28 (0.27)
Nitrogen ($g\ m^{-2}$)									

M1	0.06 (0.02;0.13)	0.53 (0.35;0.72)				0.60 (0.41;0.79)	0.40 (0.31;0.52)	0.00 (0.28)	0.00 (0.27)
M2	0.06 (0.02;0.13)	0.31 (0.11;0.53)	0.24 (0.10;0.48)			0.61 (0.43;0.80)	0.40 (0.32;0.51)	0.18 (0.28)	0.00 (0.30)
M3	0.06 (0.02;0.13)	0.48 (0.28;0.67)		0.15 (0.07;0.29)		0.70 (0.50;0.89)	0.31 (0.18;0.46)	0.07 (0.25)	0.01 (0.26)
M4	0.04 (0.00;0.12)	0.38 (0.19;0.59)			0.24 (0.10;0.45)	0.73 (0.54;0.91)	0.27 (0.15;0.41)	0.32 (0.21)	0.41 (0.25)
M5	0.06 (0.02;0.13)	0.27 (0.09;0.49)	0.22 (0.08;0.45)	0.13 (0.05;0.27)		0.70 (0.51;0.88)	0.32 (0.19;0.46)	0.19 (0.28)	0.00 (0.30)
M6	0.05 (0.00;0.13)	0.23 (0.08;0.44)	0.21 (0.08;0.42)		0.19 (0.08;0.36)	0.72 (0.53;0.91)	0.28 (0.17;0.42)	0.32 (0.25)	0.18 (0.30)
M7	0.05 (0.00;0.12)	0.22 (0.07;0.43)	0.18 (0.06;0.38)	0.10 (0.04;0.20)	0.17 (0.06;0.34)	0.76 (0.58;0.93)	0.26 (0.16;0.40)	0.34 (0.25)	0.17 (0.30)

[a]Average over 200 5-fold CVs

[b]WW: well-watered environment, WS: water-scarce environment

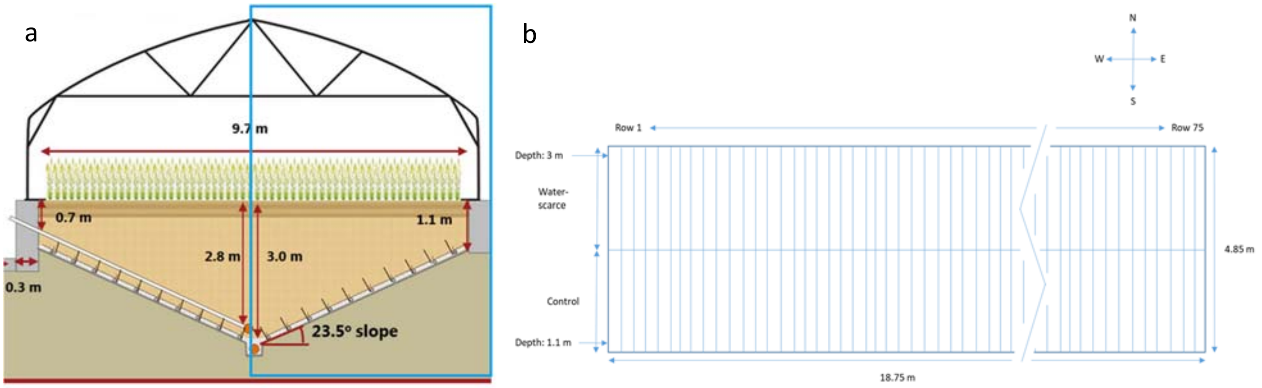

Figure S4. Cross-section of unit two and field overview of our experiment. A. Cross-section of unit two in the RadiMax facility. The experiment in this study was performed in the southside, marked with a blue square. Illustration taken from Svane et al. [73]. B. Arieal view of the field layout of our experiment, 75 rows, one for each line. The lines were split in two to define the two treatment levels.
